# Lack of Clinical Manifestations in Asymptomatic Dengue Infection Is Attributed to Broad Down-Regulation and Selective Up-Regulation of Host Defence Response Genes

**DOI:** 10.1371/journal.pone.0092240

**Published:** 2014-04-11

**Authors:** Adeline S. L. Yeo, Nur Atiqah Azhar, Wanyi Yeow, C. Conover Talbot, Mohammad Asif Khan, Esaki M. Shankar, Anusyah Rathakrishnan, Azliyati Azizan, Seok Mui Wang, Siew Kim Lee, Mun Yik Fong, Rishya Manikam, Shamala Devi Sekaran

**Affiliations:** 1 Department of Medical Microbiology, Faculty of Medicine, University of Malaya, Lembah Pantai, Kuala Lumpur, Malaysia; 2 Perdana University Graduate School of Medicine & Centre for Bioinformatics, MARDI Complex, Jalan MAEPS Perdana, Serdang, Selangor Darul Ehsan, Malaysia; 3 Institute for Basic Biomedical Sciences, The Johns Hopkins University School of Medicine, Baltimore, Maryland, United States of America; 4 Department of Pharmacology and Molecular Sciences, The Johns Hopkins University School of Medicine, Baltimore, Maryland, United States of America; 5 Department of Global Health, College of Public Health, University of South Florida, Tampa, Florida, United States of America; 6 Institute for Medical Molecular Biotechnology, Universiti Teknologi MARA, Sungai Buloh Campus, Jalan Hospital, Sungai Buloh, Selangor, Malaysia; 7 Department of Parasitology, Faculty of Medicine, University of Malaya, Lembah Pantai, Kuala Lumpur, Malaysia; 8 Department of Trauma and Emergency Medicine, University of Malaya Medical Centre, Lembah Pantai, Kuala Lumpur, Malaysia; French National Centre for Scientific Research, France

## Abstract

**Objectives:**

Dengue represents one of the most serious life-threatening vector-borne infectious diseases that afflicts approximately 50 million people across the globe annually. Whilst symptomatic infections are frequently reported, asymptomatic dengue remains largely unnoticed. Therefore, we sought to investigate the immune correlates conferring protection to individuals that remain clinically asymptomatic.

**Methods:**

We determined the levels of neutralizing antibodies (nAbs) and gene expression profiles of host immune factors in individuals with asymptomatic infections, and whose cognate household members showed symptoms consistent to clinical dengue infection.

**Results:**

We observed broad down-regulation of host defense response (innate, adaptive and matrix metalloprotease) genes in asymptomatic individuals as against symptomatic patients, with selective up-regulation of distinct genes that have been associated with protection. Selected down-regulated genes include: TNF α (*TNF*), *IL8*, *C1S*, factor B (*CFB*), *IL2*, *IL3*, *IL4*, *IL5*, *IL8*, *IL9*, *IL10* and *IL13*, *CD80*, *CD28*, and *IL18*, *MMP8*, *MMP10*, *MMP12*, *MMP15*, *MMP16*, and *MMP24*. Selected up-regulated genes include: RANTES (*CCL5*), MIP-1α (*CCL3L1/CCL3L3*), MIP-1β (*CCL4L1*), TGFβ (*TGFB*), and *TIMP1*.

**Conclusion:**

Our findings highlight the potential association of certain host genes conferring protection against clinical dengue. These data are valuable to better explore the mysteries behind the hitherto poorly understood immunopathogenesis of subclinical dengue infection.

## Introduction

Dengue represents one of the world's most dreadful vector-borne flavivirus infections with its increasing incidence, making it a major disease burden in the tropics and subtropics. Global estimates show that ∼3.6 billion individuals, representing ∼55% of the world's population, are at increasing risk for contracting dengue virus (DENV) infection. Annual estimates show that the number of dengue cases reported is ∼390 million, of which ∼96 million represent dengue hemorrhagic fever (DHF) or dengue shock syndrome (DSS), and ∼300 million others representing mild or asymptomatic cases [1]. DENV is a member of the family *Flaviviridae* and genus *Flavivirus*, a positive-stranded RNA virus transmitted by *Aedes aegypti* (*A.aegypti*) and *A. albopictus* mosquitoes. There are four serotypes namely DENV 1, 2, 3 and 4, which can all elicit the complete spectrum of disease severity, from the most common asymptomatic subclinical infection to severe plasma leakage, shock, haemorrhage and, in some cases, death. The viruses are maintained in an *A. aegypti*-human-*A. aegypti* cycle, where humans acquire infection following the bite of a DENV-infected female mosquito. The mosquito reportedly feeds on multiple individuals over a given period of time. It is, thus, common that the same mosquito could infect several members of the same household [2]–[4].

The recent WHO classification has categorized the disease as dengue without warning signs (DWOS), dengue with warning signs (DWWS) and severe dengue (SD) [5]. A DWOS DENV infection could manifest as asymptomatic or a ‘flu-like syndrome’, while DWWS could be characterized by sudden onset of fever, usually accompanied by nonspecific signs and symptoms, such as headache, back pain, stiffness, and flushed facial skin [6]. In severe dengue infections, for instance DHF (DWWS), plasma leakage and thrombocytopenia can be life threatening, especially following hypovolemic shock in severe dengue. Few studies of asymptomatic dengue have been performed and thus knowledge on the full burden of dengue infection is limited [7]. Primary DENV infections are often asymptomatic and will generate immunity to the homologous strain. However, ∼90% of DWWS (DHF) reportedly occurs following second exposure to a heterologous strain of DENV [8]. This will greatly increase the future risk of onset of SD following asymptomatic infections as the previous DENV infection had gone undetected. Therefore, subclinical infections provide ample opportunities for researchers to explore host immune factors that confer protection against clinical DENV infections.

Viral virulence [9], host genetic background [10], T-cell activation [11], viral burden [12], antibody dependent enhancement [13] and autoantibodies [14] are reportedly implicated in disease pathogenesis. Host immune factors [15]–[20] have also been reported as contributing to onset of DENV infection. It is hypothesized that DENV infection of monocytes/macrophages increases T-cell activation leading to release of cytokines and chemical mediators resulting in increased vascular permeability, plasma leakage, shock, and malfunction of the coagulatory system, culminating in hemorrhage and shock. Evidence that implicates immune factors in dengue severity is derived from chemical mediators, such as tumor necrosis factors (*TNF*), interleukin-1 (*IL1*), *IL2*, *IL6*, platelet-activating factor (PAF), complement components C3a and C5a, and histamine [21]. CD4+ T cells produce a plethora of cytokines, which recruit numerous other cytokines and chemical mediators that further increase vascular permeability [22]. Nonetheless, these studies have all concentrated on the apparent clinical dengue infection but not on asymptomatic cases, which therefore, remains a grey area of investigation. Here, we sought to investigate the molecular mechanisms underlying asymptomatic DENV infection by determining neutralizing antibody (nAb) levels and analyzing immunological genes expression profiles.

## Materials and Methods

### Specimens

Blood specimens were collected from clinical dengue cases admitted to University Malaya Medical Center (UMMC), Ampang Hospital and Klang Tengku Ampuan Rahimah Hospital, Malaysia. Blood specimens were also collected from household members of individuals presenting with clinical dengue for the investigation. The study protocols were approved by the institutional review board of the University of Malaya Medical Center (FPU-DOF-BK-012-05-R01) and from both Ampang and Klang Hospitals (Ethics no. NMRR-10-683-6420). Written informed consent from patients and asymptomatic donors was obtained, and the study was conducted in accordance with the Declaration of Helsinki. At least 1 household member's blood was collected per dengue patient. Clinical information and medical history for each patient and the accompanying household member was documented. Blood samples of dengue patients were collected during both the acute and convalescence stage. In contrast, only one time point of blood sampling was done for the suspected asymptomatic household member.

### Peripheral blood mononuclear cells

All blood samples were collected in BD Vacutainer (BD, Franklin Lakes, NJ, USA) tubes for extraction of serum. For the isolation of peripheral blood mononuclear cells (PBMCs), blood samples were collected in Vacutainer Plus (Plastic) Sterile Evacuated K2 EDTA spray dried Blood Collection Tubes (BD, Plymouth, UK). Isolation of PBMC was done using the Ficoll-Hypaque (Lymphoprep; Axis-Shield, Oslo, Norway) density gradient centrifugation [23]. Briefly, the blood was centrifuged at 2500 rpm for 15 min at 4°C; plasma was separated from the whole blood and stored at −20°C. Blood cells were washed with RPMI1640 (Roswell Park Memorial Institute) media and were gently layered over Ficoll in 50 ml centrifuge tubes. This was followed by centrifugation at 2000 rpm for 20 min without brake to separate the buffy coat from red cells. The buffy coat containing PMBCs was collected and washed with RPMI and the total number of PMBCs was determined using a hemocytometer. The isolated PBMCs were stored in liquid nitrogen until further use.

### Diagnosis of dengue

Sera from symptomatic cases and their corresponding household members were collected and subjected to *in-house* IgM-Capture ELISA [24] for IgM detection, hemagglutination inhibition (HI) test [25] for total dengue antibody detection, and quantitative real-time (qRT-PCR) [26] for detection of viral RNA. Samples were considered as confirmed dengue positive based on the criteria that any of the diagnostic assays carried out showed positivity, that is: 1) dengue nucleic acid detection through PCR, 2) IgM detection during both acute and convalescence phases, 3) sero-conversion, or 4) 4-fold increase in HI titre from acute to convalescence phase. As for the presumptive dengue positive cases, samples that showed IgM detection only during the acute phase, HI titres of more than 1280 in a single serum, or >1∶640 nAb titres of 50% reduction in neutralization by the plaque reduction neutralization test (PRNT) were considered [5]. PRNT was performed to determine the levels of neutralizing antibodies (nAb) against each of the dengue virus serotypes, following a protocol slightly modified than others [27]. Briefly, porcine kidney epithelial cells (PS cells) were seeded in 24-well plates and incubated overnight. Serum samples were diluted to 1∶10 dilution and heat-inactivated at 56°C for 30 min, followed by four-fold serial dilution using L-15 media containing 1% heat-inactivated fetal bovine serum (FBS). Equal volumes of virus with PFU of 15–30 per well were added to the diluted sera, and incubated at 37°C for 1 hour. Prototype strains of dengue viruses were used and these included Den1-Hawaiian, Den2-New Guinea C, Den3-H87 and Den4-H241. Subsequently, the virus-antibody mixture was added onto the cell monolayers in the 24-well plates, and incubated at 37°C for 3 hours. An overlay medium of 3% carboxymethyl-cellulose (CMC) was then added to the monolayers and incubated at 37°C without CO_2_ for 7 days, after which cells were washed and stained with 1% naphthalene black. The viral plaques that formed were enumerated and plaque neutralization titers were determined accordingly.

### Sample selection

Twenty-nine pairs of patients and their accompanying asymptomatic household members were recruited for gene expression investigations based on positive results for DENV infection (see the diagnosis section). Notably, among the 29 pairs, there were four that corresponded to two distinct patients paired with two asymptomatic household members each. In further microarray experiments, only the acute blood samples of the 29 patient and accompanying household member pairs were tested.

### Microarray hybridization

The stored PBMCs of patients and asymptomatic subjects were subjected to microarray hybridization (Miltenyi Biotec GmbH) as described in the manufacturer's instructions. Briefly, RNA was isolated using standard RNA extraction protocols (NucleoSpin RNA II, Macherey-Nagel). RNA samples quality was determined by the Agilent 2100 Bioanalyzer platform (Agilent Technologies). The results were visualized in a gel image and electropherogram using the Agilent 2100 Bioanalyzer expert software. The RNA integrity number (RIN) and the overall quality of total RNA were determined using the same software. RNA with RIN value >6 was subjected to linear T7-based RNA amplification to obtain sufficient antisense RNA. Amplified RNA (aRNA) was again examined on the Agilent 2100 Bioanalyzer platform, and the samples subjected to fluorescent labeling according to PIQOR user manual. The fluorescent-labeled samples were hybridized overnight to human antisense topic-defined PIQOR Immunology Microarrays (http://www.ncbi.nlm.nih.gov/geo/query/acc.cgi?acc=GPL17653) using the a-Hyb Hybridization Station. Each dengue patient-asymptomatic contact pair's RNA samples were hybridized with each other. Fluorescence signals of the hybridized PIQOR Microarrays were detected using a laser scanner (Agilent Technologies). ImaGene software (BioDiscovery, Hawthorne CA, USA) was used to obtain signal and local background intensities for each spot of the microarray images. These data are available in NCBI's Gene Expression Omnibus (GEO) database through GEO accession ID GSE50634 (http://www.ncbi.nlm.nih.gov/geo/query/acc.cgi?acc=GSE50634).

### Microarray data processing and statistical analysis

The median signal and median local background intensity values for each spot of the microarray images (obtained from ImaGene) were imported into Partek Genomics Suite (Partek Inc., St Louis MO, USA) for statistical analysis. Background subtraction was performed using the median intensities of the arrays' four replicate spots for each gene. These signal values were normalized by shifting their minimum to 2 and then converted to log2 notation for statistical analysis. Quality control of the results, using principal component analysis (PCA) and supervised hierarchical clustering (data not shown), revealed two samples to be extreme outliers so they and their paired household members' were excluded from further analysis. The paired sample t-test was used to analyze the remaining 27 household pairs, comparing the asymptomatic member's gene expression to that of the patient's.

**Figure 1:**
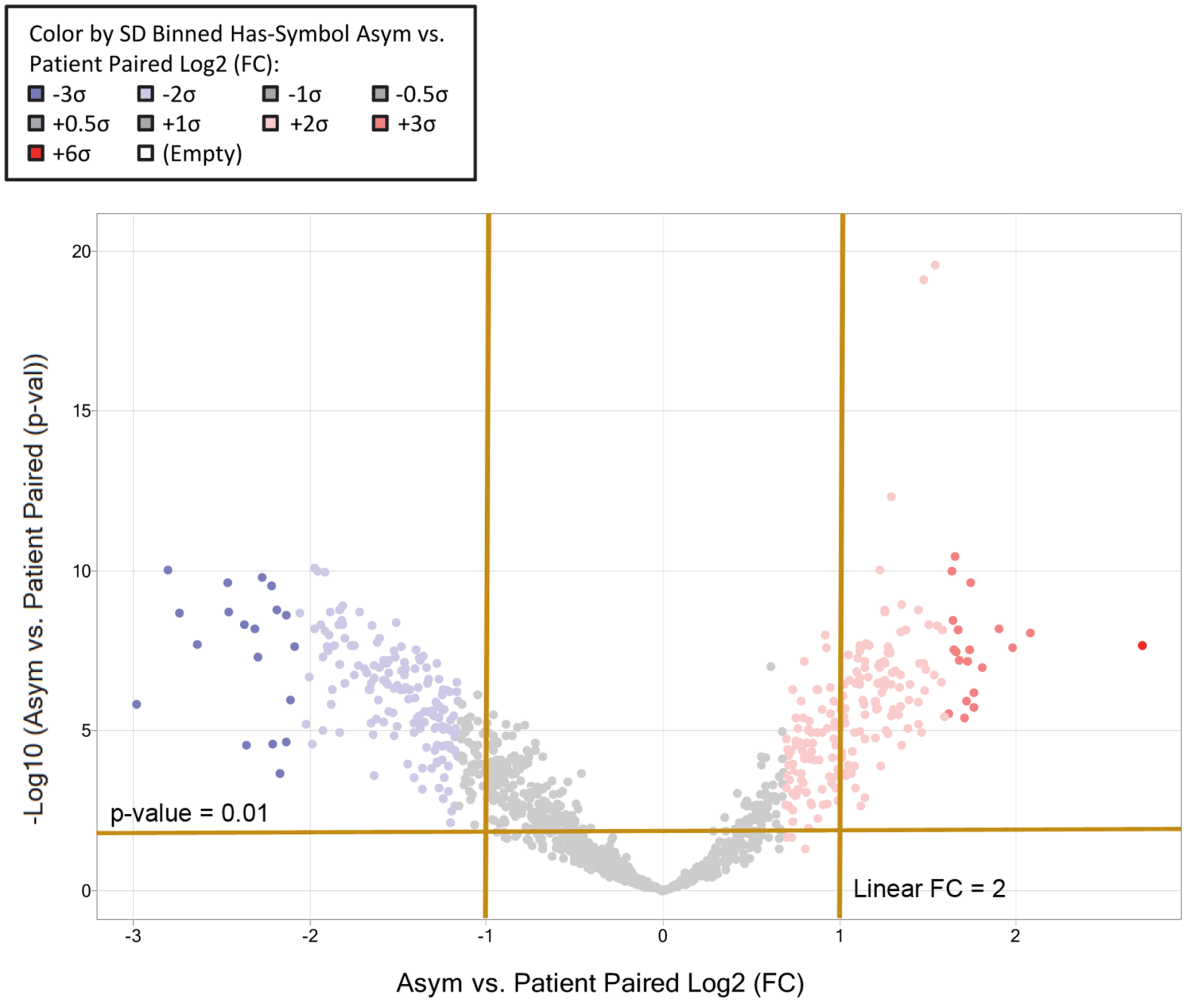
Asymptomatic versus patient paired volcano plot to determine differentially expressed genes. The *x*-axis shows the paired-sample t-test log_2_ fold change of asymptomatic versus symptomatic patients, whereas the *y*-axis shows the −log_10_ of the paired t-test p-values of asymptomatic versus symptomatic patients. Each dot represents a gene and the dot color depicts the gene's expression as shown by the standard deviation (SD) of the paired t-test log_2_ fold change, e.g. shades of blue for less than or equal to −1 SD, grey for between −1 SD and +1 SD, and red for greater than or equal to +1 SD. A total of 345 genes were differentially expressed at more than 2 and less than −2 linear fold changes (p<0.01) and were considered statistically significant.

**Figure 2:**
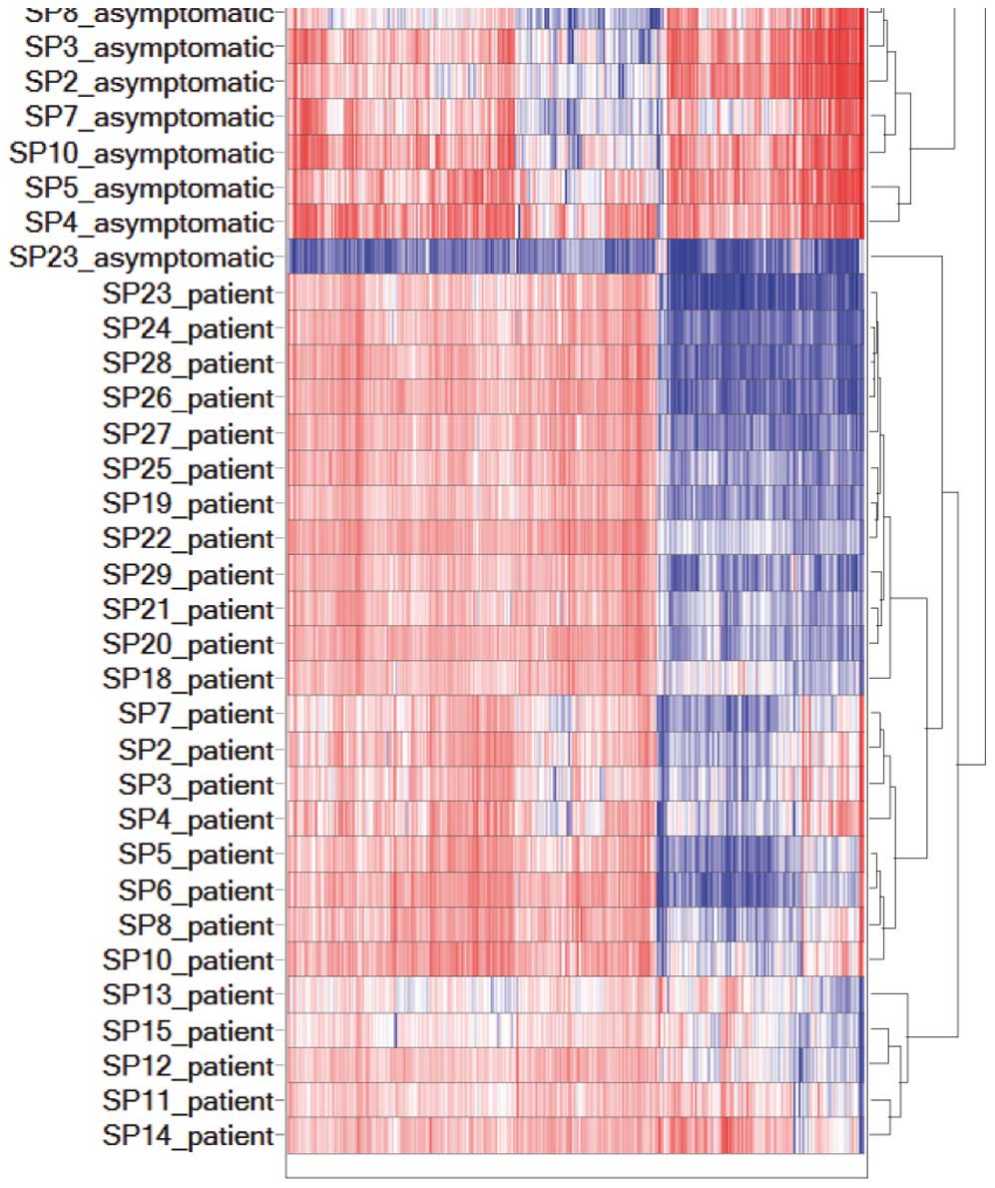
Supervised hierarchical correlation clustering of 27 sample pairs. The heat map shows the gene expression of samples in columns, with a dendogram representing their similarity based on correlation. The samples' mean-subtracted log2 signal values were clustered using complete linkage (maximum) method and the genes' expression is shown with blue representing down-regulation, white for no change (zero), and red for up-regulation.

The t-test's fold change and statistical p-values were then imported into Spotfire DecisionSite with Functional Genomics (TIBCO Spotfire Boston MA, USA) for further analysis and graphical representation. The volcano plot in Figure 1 depicts the paired sample t-test results, showing each gene's asymptomatic to symptomatic fold changes and that comparisons' statistical significance. The heat map in Figure 2 depicts a supervised clustering of all 27 samples' mean-subtracted log2 signal values for genes that were differentially expressed at more than 2 and less than −2 linear fold changes (p<0.01).

### Pathway enrichment analysis

The Spotfire DecisionSite-processed data was uploaded to Ingenuity Pathway Analysis (IPA) software for pathway enrichment analysis in order to understand their biological meaning. Ingenuity Knowledge Base (Genes Only) served as reference set for p-value calculation and Fisher's exact test was used to calculate the p-values for the canonical pathways. Only human genes and relationships were used from the database, and only experimentally observed or high confidence (predicted) observations were considered in the analysis. In addition, genes which were differentially expressed at more than 2 and less than −2 linear fold changes (p<0.01) were selected, resulting in 334 analysis-ready genes. The analysis produced a total number of 337 canonical pathways (data not shown). Among the canonical pathways, those specific to other diseases, not directly known to be relevant to dengue immune-pathogenesis/-protection, or general/unrelated signalling pathways were eliminated, leaving 53 canonical pathways for detailed analysis. The 53 selected canonical pathways were then classified into three groups, namely. innate immune response, adaptive immune response and matrix metalloproteases. Individual pathways of the innate immune response and adaptive immune response groups were studied and combined into a single mega pathway for each, whereas the matrix metalloproteases group pathway was used as-is as it was the only pathway in the group. Both the innate and adaptive mega pathways included numerous cytokines and chemokines, which are important for mediating communication among immune cells, and thus were also studied as a separate mega pathway.

## Results

### Patient characteristics

As shown in Table 1, we obtained 30 confirmed dengue positive cases of which 29 were dengue patients while 1 individual was an asymptomatic household member. There were 28 presumptive positive dengue samples of which all are asymptomatic household members. From the summary of diagnostics in Table 2, the confirmed dengue patients showed only 2 (7%) positive results for viral RNA detection. However, we found one (3%) asymptomatic household member with positive detection in PCR for viral RNA which confirmed dengue positivity. Our results showed 28 (97%) patients with positive paired IgM or seroconversion and 13 (45%) patients with HI titer >1∶1280. For presumptive positive dengue, we observed 18 (62%) asymptomatic household members with positive IgM in a single sample and 10 (34%) of these individuals had HI titre >1280. Additionally, for the PRNT that was performed, our investigations showed 9 (31%) patients and 11 (37.9%) asymptomatic individuals had nAbs against one DENV serotype as shown in Table 3. We observed dengue patients had a higher percentage (65.5%) of nAbs for polytypic infection in comparison with asymptomatic individuals (48.3%). We also observed that almost half of the asymptomatic individuals exhibited polytypic nAbs towards DENV infection.

**Table 1:** Diagnostic results of dengue patients and their accompanying household members.

Family	Identification	Infection status		Patient's sample	Presumptive positive dengue			Neutralizing antibodies			
		Confirmed dengue positive	Presumptive positive dengue		PCR	IgM	HI	Dengue 1	Dengue 2	Dengue 3	Dengue 4
1	P1	√		P1a	Negative	Negative	160	80	<10	<10	<10
				P1c		Positive	640	320	<10	<10	<10
	H1		√	H1	Negative	Positive	640	<10	320	<10	<10
2	P2	√		P2a	Negative	Positive	320	160	20	<10	<10
				P2c		Positive	640	320	<10	<10	<10
	H2		√	H2	Negative	Negative	640	<10	80	320	2560
3	P3	√		P3a	Negative	Positive	640	80	20	<10	<10
				P3c		Positive	1280	160	40	<10	<10
	H3		√	H3	Negative	Negative	640	>640	<10	<10	<10
4	P4	√		P4a	Negative	Negative	40	<10	<10	<10	<10
				P4c		Positive	160	<10	20	<10	<10
	H4		√	H4	Negative	Negative	2560	640	<10	<10	<10
5	P5	√		P5a	Negative	Positive	640	160	20	<10	<10
				P5c		Positive	2560	1280	<10	<10	<10
	H5		√	H5	Negative	Positive	5120	320	<10	<10	<10
6	P6	√		P6a	Negative	Positive	320	640	<10	<10	<10
				P6c		Positive	>10240	5120	<10	20	<10
	H6		√	H6	Negative	Positive	640	640	<10	<10	<10
7	P7	√		P7a	Negative	Negative	2560	640	40	<10	<10
				P7c		Negative	>10240	>10240	80	<10	<10
	H7		√	H7	Negative	Positive	640	320	<10	40	<10
8	P8	√		P8a	Negative	Negative	2560	2560	40	>10240	20
				P8c		Positive	>10240	>10240	80	>10240	20
	H8		√	H8	Negative	Negative	2560	320	20	<10	<10
9	P9	√		P9a	Negative	Positive	>10240	2560	<10	<10	<10
				P9c		Positive	>10240	5120	20	<10	<10
	H9		√	H9	Negative	Negative	5120	<10	<10	<10	160
10	P10	√		P10a	Negative	Negative	1280	160	<10	<10	<10
				P10c		Positive	>10240	640	<10	<10	<10
	H10		√	H10	Negative	Negative	2560	<10	<10	<10	80
11	P11	√		P11a	Dengue 1	Negative	<10	160	640	640	160
				P11c		Positive	1280	160	>2560	>2560	>2560
	H11		√	H11	Negative	Positive	>10240	>2560	>2560	>2560	>2560
12	P12	√		P12a	Negative	Positive	160	160	>2560	>2560	2560
				P12c		Positive	>10240	160	>2560	>2560	>2560
	H12		√	H12	Negative	Positive	<10	40	160	10	<10
13	P13	√		P13a	Negative	Positive	<10	160	40	2560	2560
				P13c		Positive	<10	640	640	>2560	>2560
	H13		√	H13	Negative	Positive	<10	160	<10	<10	<10
14	P14	√		P14a	Negative	Positive	20	640	160	40	160
				P14c		Positive	>10240	>2560	>2560	160	2560
	H14		√	H14	Negative	Negative	10	640	<10	40	<10
15	P15	√		P15a	Negative	Positive	20	640	160	40	160
				P15c		Positive	>10240	>2560	>2560	160	2560
	H15		√	H15	Negative	Negative	80	<10	<10	<10	<10
16	P16	√		P16a	Negative	Positive	1280	<10	<10	40	<10
				P16c		Positive	>10240	<10	<10	2560	<10
	H16		√	H16	Negative	Positive	20	<10	<10	<10	<10
17	P17	√		P17a	Negative	Positive	1280	<10	640	<10	<10
				P17c		Positive	2560	<10	1280	<10	<10
	H17		√	H17	Negative	Positive	10	<10	<10	<10	<10
18	P18	√		P18a	Negative	Positive	10	<10	<10	<10	<10
				P18c		Positive	160	160	<10	<10	<10
	H18		√	H18	Negative	Positive	10	<10	<10	<10	<10
19	P19	√		P19a	Negative	Positive	>10240	>2560	160	640	<10
				P19c		Positive	>10240	>2560	>2560	2560	160
	H19		√	H19	Negative	Positive	1280	40	40	160	40
20	P20	√		P20a	Negative	Positive	640	160	640	<10	<10
				P20c		Positive	2560	160	640	<10	<10
	H20		√	H20	Negative	Positive	640	<10	160	<10	<10
21	P21	√		P21a	Negative	Positive	2560	640	160	40	640
				P21c		Positive	2560	640	160	40	640
	H21	√		H21	Dengue 3	Negative	320	160	160	640	160
22	P22	√		P22a	Negative	Positive	2560	640	160	40	640
				P22c		Positive	2560	640	160	40	640
	H22		√	H22	Negative	Positive	10	<10	<10	40	<10
23	P23	√		P23a	Negative	Positive	5120	40	2560	10	40
				P23c		Positive	>10240	640	2560	10	40
	H23		√	H23	Negative	Equivocal	10240	10	160	10	10
24	P24	√		P24a	Negative	Positive	20	<10	10	<10	<10
				P24c		Positive	40	<10	40	<10	<10
	H24		√	H24	Negative	Positive	320	40	40	40	10
25	P25	√		P25a	Negative	Positive	2560	<10	640	640	<10
				P25c		Positive	>10240	<10	640	2560	<10
	H25		√	H25	Negative	Negative	160	<10	40	<10	<10
26	P26	√		P26a	Dengue 3	Positive	160	<10	<10	160	<10
				P26c		Positive	1280	<10	<10	640	<10
	H26		√	H26	Negative	Positive	5120	<10	2560	1280	<10
27	P27	√		P27a	Negative	Positive	1280	40	1280	10	<10
				P27c		Positive	10240	2560	1280	10	<10
	H27		√	H27	Negative	Negative	640	<10	40	10	<10
28	P28	√		P28a	Negative	Positive	1280	40	640	160	40
				P28c		Positive	5120	2560	640	160	40
	H28		√	H28	Negative	Equivocal	80	10	10	10	10
29	P29	√		P29a	Negative	Negative	<10	<10	10	10	10
				P29c		Positive	10	160	10	10	10
	H29		√	H29	Negative	Positive	2560	40	1280	640	40

*P indicates dengue patient while H indicate household member.

**a indicates acute samples while c indicates convalescence sample.

**Table 2:** Summary of dengue diagnostic results obtained from patients and their corresponding/accompanying household members.

Diagnostic test	Patients (%)	Household members (%)
Real time RT-PCR	2 (7)	1 (3)
Positive IgM	28 (97)	18 (62)
HI>1280	13 (45)	10 (34)

**Table 3:** Summary of plaque reduction neutralization test (PRNT) results obtained from patients and their corresponding/accompanying household members.

Category	Monotypic Infection	Polytypic Infection	No neutralizing antibody detected	Total
Dengue patient acute sera	9 (31.0)	19 (65.5)	1 (3.4)	29
Asymptomatic dengue individuals	11 (37.9)	14 (48.3)	4 (13.8)	29

The numbers within parentheses indicate percentage.

### Differentially expressed genes determined from paired-sample t-test

The volcano plot, Figure 1 displays relative gene expression, showing the standard deviation (SD) of the paired t-test log_2_ fold change against −log_10_ p-value of the paired t-test. The high statistical significance of genes as depicted in this plot provided confidence in reliability of the expression values. A total of 345 genes were differentially expressed at more than 2 and less than −2 linear fold changes (p<0.01) and were considered statistically significant.

### Supervised hierarchical clustering discriminates symptomatic patients from asymptomatic individuals

The hierarchical supervised correlation clustering of 27 sample pairs (SPs), Figure 2, shows that a majority of the patients clustered together as they had similar gene expression patterns, except for SP16 and SP17 patients who separated the asymptomatic household members into two clusters. This separation, however, was not considered significant as it was attributed to batch effect as suggested by the PCA plot (data not shown). SP23_asymptomatic, which clustered by itself in a single group, was not deemed as an outlier according to the PCA plot and thus was retained for further analysis. Based on the overall clustering, it is clear that the gene expression patterns of the asymptomatic household members' were different from the patients'.

### Gene profiling for biological significance

Pathway enrichment analysis of the 27 paired sample data was performed to glean the biological significance of the differential expression pattern observed between asymptomatic individuals and symptomatic patients. The resulting 53 selected pathways with their respective expressed genes shown in Table 4 (see Table S1 for more details) were grouped according to the two broad categories of reported dengue host defence mechanisms, namely innate immune response (Figure S1) and adaptive immune response (Figure S2). Additionally, the global role of the cytokines and chemokines, which mediate communication between the immune cells, was studied through a separate pathway in Figure S3. The matrix metalloproteases pathway was studied as well and shown in Figure S4.

**Table 4:** 53 of the selected canonical pathways studied, with genes up- and down-regulated in the subclinical/asymptomatic dengue are indicated.

Group	Canonical Pathway		Differential Expression	
	No.	Name	Up-regulation	Down-regulation
Innate	1	Acute phase response Signaling	*AKT1, FOS, IKBKG, NFKB1, NFKBIA, TRADD*	*C1S, CFB, CRP, IL1A, IL1RAP, MAP2K7, SOCS3, TNF*
	2	*CCR5* signaling in macrophages	*CALM1, CCL5, CD247, CD3D, CD3E, FOS, PRKCB*	*FASLG, PLCG1, PRKCE*
	3	*CD27* signaling in lymphocytes	*FOS, IKBKG, NFKB1, NFKBIA*	*MAP2K7, MAP3K2, MAP3K4*
	4	*CD40* signaling	*FOS, IKBKG, NFKB1, NFKBIA*	*ICAM1, LTA, MAP2K7, PIK3C2A, PIK3C2G*
	5	Complement system	*-*	*C1S, CFB*
	6	Crosstalk between dendritic cells and natural killer cells	*ACTB, ICAM3, IL2RG, KLRC4-KLRK1/KLRK1, KLRD1, LTB, NFKB1, PRF1*	*CD28, CD80, CSF2, CSF2RB, FASLG, IL2, IL2RB, IL3, IL4, LTA, LTBR, TNF*
	7	Dendritic cell maturation	*AKT1, B2M, CD1D, FCGR3A, HLA-DQA1, IKBKG, LTB, NFKB1, NFKBIA*	*CD1A, CD1B, CD80, COL11A2, COL1A1, COL1A2, COL2A1, COL3A1, CSF2, ICAM1, IL10, IL1A, LTA, LTBR, LY75, PIK3C2A, PIK3C2G, PLCB1, PLCB4, PLCD1, PLCD3, PLCE1, PLCG1, TNF*
	8	iNOS signaling	*CALM1, FOS, IKBKG, IRF1, NFKB1, NFKBIA*	*-*
	9	Mechanisms of viral exit from host cells	*ACTB, PRKCB*	*NEDD4, PRKCE*
	10	*MIF* regulation of innate immunity	*CD74, FOS, NFKB1, NFKBIA*	*PLA2G12B, PLA2G3, PLA2G4A*
	11	Natural killer cell signaling	*AKT1, CD247, FCGR3A, FYN, KLRC4-KLRK1/KLRK1, KLRD1, LCK, PRKCB*	*PIK3C2A, PIK3C2G, PLCG1, PRKCE, VAV3*
	12	Production of Nitric Oxide and reactive Oxygen species in macrophages	*AKT1, CLU, FOS, IKBKG, IRF1, NFKB1, NFKBIA, PRKCB, RHOA*	*IL4, MAP2K7, MAP3K2, MAP3K4, PIK3C2A, PIK3C2G, PLCG1, PPARA, PRKCE, TNF*
	13	Role of pattern recognition receptors in recognition of bacteria and viruses	*CASP1, CCL5, NFKB1, PRKCB*	*IL10, IL2, PIK3C2A, PIK3C2G, PRKCE, TLR6, TNF*
	14	Role of PKR in interferon induction and antiviral response	*AKT1, IKBKG, IRF1, NFKB1, NFKBIA*	*TNF*
	15	Role of *RIG1*-like receptors in antiviral innate immunity	*IKBKG, NFKB1, NFKBIA*	*CASP10, IFNA2*
	16	Toll-like receptor signaling	*FOS, IKBKG, NFKB1, NFKBIA*	*PPARA, TLR6*
	17	TWEAK signaling	*IKBKG, NFKB1, NFKBIA, TNFRSF25, TRADD*	*CASP7*
Adaptive	18	B cell development	*HLA-DQA1, IL7R, PTPRC*	*CD80, RAG1*
	19	B cell receptor signaling	*AKT1, CALM1, CSK, IKBKG, NFATC3, NFKB1, NFKBIA, PRKCB, PTEN, PTPRC*	*EGR1, MAP2K7, MAP3K2, MAP3K4, PIK3C2A, PIK3C2G, VAV3*
	20	B cell activating factor signaling	*FOS, IKBKG, NFATC3, NFKB1, NFKBIA*	*MAP2K7*
	21	*CD28* signaling in T helper cells	*AKT1, CALM1, CD247, CD3D, CD3E, CSK, FOS, FYN, HLA-DQA1, IKBKG, ITK, LCK, NFATC3, NFKB1, NFKBIA, PTPRC*	*CD28, CD80, IL2, PIK3C2A, PIK3C2G, PLCG1*
	22	*CTLA4* signaling in cytotoxic T lymphocytes	*AKT1, B2M, CD247, CD3D, CD3E, FYN, LCK*	*CD28, CD80, PIK3C2A, PIK3C2G, PLCG1*
	23	Cytotoxic T lymphocytes-mediated apoptosis of target cells	*B2M, CD247, CD3D, CD3E, HLA-DQA1, PRF1*	*BCL2, CASP7, FASLG*
	24	Differential regulation of cytokine production in intestinal epithelial cells by *IL17A* and *IL18*	*CCL5*	*CSF2, CXCL1, IL3, IL9, IL10, IL13, IL1A, TNF*
	25	Differential regulation of cytokine production in macrophages and T helper cells by *IL17A* and *IL18*	*CCL5*	*CSF2, CXCL1, IL3, IL9, IL10, IL13, TNF*
	26	Fcγ Receptor-mediated Phagocytosis in Macrophages and Monocytes	*ACTB, AKT1, FCGR3A, FGR, FYN, PRKCB, PTEN*	*CBL, CSF2, PIK3C2G, PLCG1, PRKCE, VAV3, YES1*
	27	FcγRIIB Signaling in B Lymphocytes	*AKT1*	*PIK3C2A, PIK3C2G*
	28	NF-kB activation by viruses	*AKT1, IKBKG, ITGB1, ITGB2, LCK, NFKB1, NFKBIA, PRKCB*	*ITGA2, ITGAV, PIK3C2A, PIK3C2G, PRKCE*
	29	T cell receptor signaling	*CALM1, CD247, CD3D, CD3E, CSK, FOS, FYN, IKBKG, ITK, LCK, NFATC3, NFKB1, NFKBIA, PTPRC*	*CBL, CD28, PIK3C2A, PIK3C2G, PLCG1, VAV3*
	30	T helper cell differentiation	*HLA-DQA1, IL10RA, IL2RG, TBX21, TGFB1*	*CD28, CD80, FOXP3, IL10, IL13, IL18R1, IL2, IL4, IL5, TGFBR1, TNF*
Cytokine	31	Chemokine signaling	*CALM1, CCL5, CXCR4, FOS, MPRIP, PRKCB, RHOA*	*PIK3C2G, PLCB1, PLCB4, PLCG1*
	32	GM-CSF signaling	*AKT1, PRKCB*	*CCND1, CSF2, CSF2RA, CSF2RB, PIK3C2A, PIK3C2G*
	33	*IL1* signaling	*FOS, IKBKG, NFKB1, NFKBIA*	*ADCY8, IL1A, IL1RAP, MAP2K7*
	34	*IL10* signaling	*FOS, IKBKG, IL10RA, NFKB1, NFKBIA*	*IL10, IL1A, IL1RAP, IL1RAPL1, IL1RAPL2, SOCS3, TNF*
	35	*IL15* production	*IRF1, NFKB1*	*MST1R*
	36	*IL15* signaling	*AKT1, IL2RG, LCK, NFKB1, STAT5A*	*BCL2, CSF2, IL2RB, IL4, PIK3C2A, PIK3C2G, PLCG1*
	37	*IL2* signaling	*AKT1, FOS, IL2RG, LCK, STAT5A*	*IL2, IL2RB, PIK3C2A, PIK3C2G*
	38	*IL22* signaling	*AKT1, STAT5A*	*IL22RA1, SOCS3*
	39	*IL3* signaling	*AKT1, FOS, PRKCB, STAT5A*	*CRKL, CSF2RB, IL3, PIK3C2A, PIK3C2G, PRKCE*
	40	*IL4* signaling	*AKT1, HLA-DQA1, IL2RG, NFATC3*	*IL4, PIK3C2A, PIK3C2G*
	41	*IL6* signaling	*AKT1, FOS, IKBKG, NFKB1, NFKBIA*	*COL1A1, CRP, IL1A, IL1RAP, IL1RAPL1, IL1RAPL2, IL8, MAP2K7, PIK3C2A, PIK3C2G, SOCS3, SRF, TNF*
	42	*IL8* signaling	*AKT1, FOS, IKBKG, ITGAX, ITGB2, NFKB1, PRKCB, RHOA*	*BCL2, CCND1, CXCL1, FIGF, ICAM1, IL8, IL9, ITGAV, KDR, PGF, PIK3C2A, PIK3C2G, PRKCE, VEGFC*
	43	*IL9* signaling	*IL2RG, NFKB1, STAT5A*	*IL9, PIK3C2A, PIK3C2G, SOCS3, TNF*
MMP	44	Inhibition of matrix metalloproteases	*TIMP1*	*MMP10, MMP12, MMP15, MMP16, MMP24, MMP8*
Interaction between innate, cytokines and adaptive	45	*TREM1* signaling	*AKT1, CASP1, ITGAX, ITGB1, NFKB1, STAT5A*	*CSF2, CXCL3, ICAM1, IL10, IL8, PLCG1, TLR6, TNF*
	46	Interferon signaling	*IRF1, PTPN2, TAP1*	*BCL2, IFNAR2*
	47	*IL17* signaling	*AKT1, IL17RA, NFKB1, TIMP1*	*CRP, CXCL1, IL19, IL8, PIK3C2A, PIK3C2G*
	48	*IL17A* signaling in fibroblasts	*FOS, IKBKG, IL17RA, NFKB1, NFKBIA*	*-*
	49	Regulation of *IL2* expression in activated and anergic T lymphocytes	*CALM1, CD247, CD3D, CD3E, FOS, FYN, IKBKG, NFATC3, NFKB1, NFKBIA, TGFB1*	*CD28, CD80, IL2, MAP2K7, PLCG1, TGFB2, TGFBR1, VAV3*
	50	TGF-β Signaling	*BMP4, FOS, TGFB1*	*BCL2, BMP2, BMP7, FOXH1, PIAS4, TGFB2, TGFBR1*
	51	Communication between innate and adaptive immune cells	*B2M, CCL3L1/CCL3L3, CCL5*	*CD28, CD80, CSF2, IL10, IL1A, IL2, IL3, IL4, IL5, IL8, TLR6, TNF*
	52	*IL12* signaling and production in macrophages	*AKT1, CLU, FOS, IKBKG, IRF1, NFKB1, PRKCB, TGFB1*	*IFNA2, IL10, IL4, MST1R, PIK3C2A, PIK3C2G, PRKCE, TGFB2, TNF*
	53	Role of cytokines in mediating communication between immune cells	*TGFB1*	*CSF2, IFNA2, IL10, IL13, IL1A, IL2, IL3, IL4, IL5, IL8, TNF*

The pathways have been grouped according to the broad categories of reported dengue host defence mechanisms. The gene symbols are based on HUGO Gene Nomenclature Committee (HGNC) symbols. See Table S1 for more details as it also includes the list of genes that showed no change for each of the pathways listed and provides linear fold change for genes discussed in the manuscript.

#### Innate immune response

The expression of Toll-like receptors (TLRs), which are critical for pathogen recognition, was observed (Table 4; Figure S1, and S5 for pathway legends). The TLR complex indicated in the figure includes several genes (*TLR2*, *TLR3*, *TLR4*, *TLR7* and *TLR9*) that remained unchanged between asymptomatic household members and symptomatic patients, and one (*TLR6*) that was down-regulated. We also observed expression of *CD40*, *CD83* and *CD86* but at no significant level change. However, *CD80* expression was significantly down-regulated. Besides that, we observed a no change in CD32A (*FCGR2B*), but an up-regulation in CD16A (*FCGR3A*). Regarding the complement system, we observed down regulation of *C1S* and complement factor B (*CFB*). *C1S* is a protein component that cleaves C2 and C4 into C2a, C2b, C4a and C4b in the classical pathway, while *CFB* is a component of the alternative pathway.

#### Adaptive immune response

The expression of both T-cell antigen presentation molecules MHC class 1, found on all nucleated cells, and MHC class II, expressed on B-cells, macrophages and dendritic cells, were up-regulated. However, there was no change in the expression levels of both CD8 and CD4 complexes. Meanwhile, the results showed a down-regulation of *CD28* (Table 4; Figure S2, S5), which is expressed on T cells to provide co-stimulatory signal for T-cell activation upon binding to *CD80*, which was also down-regulated (Table 4; Figure S2). We also observed an up-regulation in asymptomatic siblings for T-bet (TBX21) (Table 4; Figure S2), which is a critical regulator for T helper cell 1 (Th1) differentiation and an inducer for the production of IFNγ (IFNG) [28]. It simultaneously inhibits the opposing Th2 and Th17 differentiation program. Additionally, *FOXP3*, a transcription factor involved in regulatory T cell (Treg) development was seen to be down-regulated.

#### Cytokines and chemokines

Cytokines and chemokines are important inflammatory mediators of both innate and adaptive immune responses that are stimulated in response to pathogens. In our study, we found a down regulation of IL1α (*IL1A*), *IL2*, *IL3*, *IL4*, *IL5*, *IL8*, *IL9*, *IL10*, *IL13*, IL18R (*IL18R1*), IL22R (*IL22RA1*), TNFα (*TNF*), TGFβR (*TGFBR1*), GM-CSF (*CSF2*) and MIP-2 (*CXCL3*) expression as shown in Table 4 and Figure S3. Observed up-regulated genes included MIP1α (*CCL3L1/CCL3L3*), MIP1β (*CCL4L1*), RANTES (*CCL5*), TGFβ1 (*TGFB1*), IL4R IL10R (*IL10RA*) and *IL7R*. Genes with no significant change in expression level between asymptomatic siblings and symptomatic patients included IL1β (*IL1B*), *IL6*, *IL7*, IL12 (*IL12B*), *IL15*, *IL17A*, *IL17F*, *IL18*, *IL21*, *IL24*, IFNα (*IFNA1/IFNA13*), IFNβ (*IFNB1*), IFNγ (*IFNG*), *CCL2* and *CCR5*.

#### Matrix metalloproteases (MMPs)

MMPs are a family of proteins that cleave most extracellular matrix constituents. We observed the reduced expression of MT-MMP (includes *MMP15*, *MMP16*, and *MMP24*, among others) and Extracellular MMP (includes *MMP8*, *MMP10*, and *MMP12*, among others) complexes (Figure S4, S5). The genes *MMP8*, *MMP10*, *MMP12*, *MMP15*, *MMP16* and *MMP24* were down-regulated in asymptomatic siblings, while *MMP2* and *MMP9* were detected with similar expression level in both groups of subjects (Table 4; Figure S4, S5). *TIMP1*, a factor that inhibits activity of MMPs was up-regulated.

## Discussion

The differences in dengue clinical manifestations are widely being investigated to better understand the disease pathogenesis. Herein, we investigated the protective mechanisms contributing to the apparent lack of clinical manifestations in individuals that remain asymptomatic when compared to clinical dengue cases.

We note that selection criteria for gene expression study herein are based solely on the presumptive positive IgM levels and HI titres of single asymptomatic samples as there were difficulties in obtaining second voluntary blood samples from the household members. From the diagnostic results, only three subjects were PCR positive. Virus titre could not be determined through PCR for most of the samples due to the duration of infection. The samples collected for PCR were mostly from days 5 to 6, by which time viral titres may have declined, with increasing antibody levels. Thus, PRNT, a gold standard test used widely to determine and quantify nAbs against DENV infection [29], was carried out to mitigate these limitations. We observed that asymptomatic individuals had high concentration of nAbs against DENV infection. Despite having polytypic infections, half of these asymptomatic individuals are protected against clinical dengue. It has been reported that antibodies could play a greater role than immune cells in heterologous DENV infection [30]. This entails that individuals infected with DENV manifest clinical symptoms differently with some presenting with more severe symptoms than others due to the presence of pre-existing nAbs in the asymptomatic individuals. However, apart from nAbs, protection from DENV infection in asymptomatic individuals could also be attributed to other factors viz., host genetic factors [31], which comprise a complex network of genes that are expressed differentially in the asymptomatic individuals.

Inflammation involves the activation of immune cells and recruitment of specific immune cells to the site of inflammation [32], eventually contributing to nonspecific clearance of DENV, albeit causing mild symptoms. However, in some cases, exaggerated inflammation can culminate in detrimental clinical manifestations, pathognomonic of DWWS/SD. The mutual synergism between the innate and adaptive immune responses is the key to responding to infectious microorganisms. Here, we hypothesize that dengue asymptomatic individuals downplay the factors responsible for inflammation and regulate the associated immune factors to a level that is merely required to facilitate virus clearance.

### Genes of the innate immune response

The innate host response to DENV is mediated by dendritic cells (DCs), phagocytes (macrophages) and natural killer lymphocytes which sense viral proteins or nucleic acids through Toll-like receptors (TLRs). In our study, we observed no change in the expressions of *TLR3* and *TLR7* between the asymptomatic individuals and symptomatic patients. *TLR 3* has been shown to play an important role in restricting DENV infection in synergy with *RIG-I* and *MDA5* [33]. We could not assess this relationship due to the absence of *RIG-I* and *MDA5* from the array used. Besides, each TLR recognizes distinct microbial components and activates different signaling pathways by selective utilization of adaptor molecules. Since dengue is a single stranded RNA virus, *TLR3* may not respond as it is known to recognize viral double stranded RNA [34]. However, in support of *TLR3* as a protective factor, recent studies have demonstrated that its expression induces type I interferon and inhibits the replication of DENV in different cell lines [35], [36]. In contrast, *TLR7* receptor has been shown to recognize the virus, with significantly higher expression levels in DHF patients than in DF [37], [38]. Although this does not explain the role of *TLR7* in the protective mechanism(s) preventing the development of clinical symptoms, a no change observation in the gene may relatively reduce the effect of more severe clinical symptoms that could have resulted from an up-regulation of *TLR7*.

FcγR is critical in binding to the DENV immune complex, enhancing virus uptake by DCs and macrophages [39]. We observed a no change in CD32A (*FCGR2B*), but an up-regulation of CD16A (*FCGR3A*). These Fcγ receptors connect the innate and the adaptive immune responses by transmitting activating signals to natural killer lymphocytes and myeloid cell upon recognition of Fc of IgG [40]. CD32A is expressed in all myeloid cells, platelets, and endothelial cells, whereas CD16A is present on monocytes, macrophages, NK cells and γ/δ T cells [41]. The up-regulation of CD16A in our study suggests the activation of antibody response by the NK cells, which may be a protective factor in dengue clinical manifestation. This is supported in other studies that CD16A is the only Fc receptor expressed on NK cells and is responsible for IgG-initiated antibody dependent cell-mediated cytotoxicity (ADCC) [42], which may reduce the incidence of antibody-dependent enhancement (ADE), one of the hallmarks of dengue pathogenesis.

The complement system is an important arm of innate immunity. Earlier studies suggest that complement activation plays a role in the pathogenesis of DHF [43], [44]. We observed a down regulation of *C1S* and factor B complement expression in asymptomatic siblings. An *in vitro* study provided evidence that complements promote the uptake of DENV into myeloid cells through *CR3*, augmenting the infection [45]. Several studies have shown that DENV non-structural protein 1 (NS1) binds to C4 and *C1S*, regulating complement activation and hence could be involved in DHF pathogenesis [44], [46]. Therefore, with the decreased expression of *C1S* in our study, the activation of complement system is reduced, suppressing severe dengue pathogenesis and allowing only subclinical symptoms to be manifested.

### Genes of the adaptive immune response

The adaptive immune system includes antibody-secreting B cells and cytotoxic T cells that specifically and efficiently target the pathogen and infected cells. Activated DENV-infected antigen presenting cells (APCs), such as DCs, macrophages or B-cells, acquire an activation profile with elevated expression of *CD40*, *CD80*, *CD83* and *CD86* [47], [48]. These surface proteins assist the communication between DENV-infected cells and T cells. In the present study, we observed a down regulation of *CD80* expression in asymptomatic siblings, and detected no expression change for *CD40*, *CD83* and *CD86*. An *in vitro* study demonstrated that defect in co-stimulatory proteins failed to provide adequate signaling for T cell activation and proliferation, resulting in impaired cell-mediated response [49]. This suggests an ineffective interaction between DENV-infected immune cells and T cells, despite up-regulation of MHC I (Beta-2-microglobulin, *B2M*) and MHC II (*HLA-DQA1*) complex members' expressions. Nonetheless, there was no change in the expression levels of CD8 and CD4 T-cell complexes between both groups. This suggests that the host immune response may have reduced disease severity that is caused by T-cell-mediated tissue damage. It was reported in a study that the magnitude of T-cell responses correlates with dengue disease severity due to cross reactive T cells [50]. Moreover, the down regulation of *CD28*, required to provide the co-stimulatory signal for T-cell activation, and cytokines produced by T cells, namely *IL2*, *IL4*, *IL5*, *IL10* and *IL18*, further reinforces the broad suppression of cell-mediated immune response in asymptomatic siblings.

Naïve T helper cells can differentiate into Th1, Th2, Th17, follicular T helper cells (TFH) and T regulatory cells (Tregs) [51]. We observed an up regulation of T-bet in asymptomatic siblings. This is suggestive of a partial Th1 response, which plays a role in viral clearance but not in causing clinical symptoms. Furthermore, it has been shown that T-bet inhibits production of Th2 and Th17 cytokines [52], and with a reduced expression of IL4 in asymptomatic siblings, it is reasonable to infer that Th2 responses are suppressed by T-bet.

### Cytokines and chemokines role in protection

Cytokines and chemokines are important inflammatory mediators that are stimulated in response to pathogens and certain cytokine profiles have been associated with dengue disease severity [53]–[58]. It has been suggested that the pathogenesis of DF/DHF involves amplified cytokine production, also known as “cytokine storm” [59], [60]. This ultimately leads to excessive immune activation that increases vascular permeability [58] and causes plasma leakage and shock. Our results showed a general down regulation in expression of cytokines and chemokines, specifically for *IL2*, *IL3*, *IL4*, *IL5*, *IL8*, *IL9*, *IL10* and *IL13*. However, elevated cytokines including *IL2*, *IL4*, *IL6*, *IL8*, *IL10*, *IL13*, *IL18*, TNFα (*TNFA*), IFNγ (*IFNG*), TGFβ (*TGFB*), *CCL2* and *CCL3* have been reported in patients with DHF [53], [54], [57], [61]–[64]. This suggests a limited inflammatory response trigger, sufficient for viral clearance yet below the level that can lead to clinical symptoms. Similarly, other studies have shown some of these cytokines to serve as predictive markers for progression to dengue with warning signs when cytokine kinetics and profiles of dengue patients at different phases of illness were investigated [65].

We observed down regulation of TNFα herein, suggesting an outcome similar to healthy controls. Studies have reported a significantly higher level of TNFα in dengue-infected patients compared to healthy controls, and the elevated TNFα is associated with disease severity [66], [67]. Hence, the down regulation seen in our study's asymptomatic individuals may have been a protective factor in controlling the severity of the disease. In addition, TNFα308 allele polymorphism was observed among the DHF patients and they expressed higher levels of TNFα [68]. We also observed down regulation of IL1α, which may point to the possible role of TNFα and IL1α in dengue pathogenesis. This is supported by another study that has demonstrated TNFα and IL1α to increase vascular permeability *in vitro* [69].

DENV-infected DCs or macrophages can produce chemokines including *IL8*, RANTES (*CCL5*), *MCP-1*, *MIP1* and *IP-10*. *IL8*, a chemokine produced by stimulated monocytes is down-regulated in our study. This is consistent with a previous study that showed association of higher *IL8* level with disease severity [70]. It has been shown that *IL8* has a chemoattractant and degranulation ability for neutrophils [71]. It can also increase the permeability of the endothelial cell monolayer, as shown *in vitro* [72]. Increased endothelial cell permeability may lead to the development of plasma leakage, a clinical manifestation of severe dengue patients. Therefore, the reduced expression of *IL8* in asymptomatic siblings could contribute to their protective mechanisms in preventing clinical manifestations. RANTES is a chemokine that recruits T cells, eosinophils and basophils to the site of infection. Our results showed an up-regulation of RANTES in asymptomatic siblings. We hypothesize that high RANTES levels may play an important role in mitigating clinical dengue symptoms in infected individuals. This is because a previous study has reported that plasma RANTES levels is lower in acute dengue infants compared to infants with other febrile infections [73]. In another study, an association was observed between decreased RANTES levels in patients with acute dengue fever and lower platelet number or thrombocytopenia [74]. Comparing our results with these earlier studies, higher RANTES expression appears to be advantageous in mitigating clinical disease.

In this study, we observed an up-regulation of MIP-1β. MIP-1β has been reported to have an association with good prognosis factors identified in different disease models [75], [76]. In one study, MIP-1β levels were higher in patients with mild dengue fever compared to patients with severe clinical manifestation [77]. The authors suggested that the relationship of MIP-1β and NK cells may play a role in dengue protective mechanisms. Consistent with these findings, our results of up-regulated MIP-1β in asymptomatic siblings further reinforces the possible protective role of MIP-1β.

TGFβ is a multifunctional cytokine that can act as a proinflammatory or anti-inflammatory cytokine depending on its concentration. Our results showed a higher TGFβ expression level in asymptomatic patients, which suggests a protective role for TGFβ in dengue disease. This is supported by a study that showed a TGFβ polymorphism that demonstrated high TGFβ production was associated with protection and mild clinical manifestations [78]. However, on the contrary, there are other studies that report a significantly higher plasma level of TGFβ in DHF patients as compared to DF patients [54], [79]. Given these effects, it remains to be ascertained whether TGFβ expression contributes to a pathogenic or protective role in dengue infection.

### MMPs role in dengue infection

MMPs are a family that cleave most extracellular matrix constituents. In our study, we detected no change in *MMP9* and *MMP2* expressions between patients and asymptomatic subjects, but did observe a broad down-regulation of *MMP8*, *MMP10*, *MMP12*, *MMP15*, *MMP16* and *MMP24* expressions. Meanwhile, the inhibitor of MMPs, *TIMP1* was up-regulated, which may explain the broad down-regulation of extracellular MMPs that we observed. These findings indicated an overall suppression of MMPs, which may contribute to the protective mechanism in dengue infection. This is reasonable given a study that pointed to the role of MMP in triggering plasma leakage in DHF patients [80]. Activation of *IL6*, *TNFα*, *IL8*, and *TGFβ* can drive MMP production, which is correlated to the vascular leakage characteristics of DHF [54], [71], [81], [82]. Additionally, overexpression of *MMP9* and to a lesser extent *MMP2* were observed to have a role in enhancing vascular permeability [20]. This study suggests the role of *MMP9* and *MMP2* in dengue pathogenesis.

In summary, DENV infection among asymptomatic individuals, as compared to clinical dengue patients, provides a wealth of information associating gene expression and immune correlates. Overall, we observed broad down-regulation of host defense response (innate, adaptive, cytokines and matrix metalloprotease) genes in asymptomatic individuals against symptomatic patients, with selective up-regulation of distinct genes that have been associated with protection. Several of the genes examined herein deserve further assessment to correlate expression with conferring protection against clinical DENV infection. Given the emergence of a wider network of immune molecules in subclinical DENV infection, additional studies may be warranted to investigate the molecular targets associated with improved clinical manifestations and increase understanding of the pathogenesis of subclinical DENV infection.

## Supporting Information

Figure S1 **Innate immune response mega canonical pathway created using Ingenuity Pathway Analysis.** TLR complex includes *TLR6* (down-regulated in asymptomatic individuals), *TLR3* (no change in expression between the two groups) and *TLR7* (no change), among others. Please refer to Figure S5 for a legend explaining Pathway molecule symbols. For additional information, visit IPA legend help page at http://ingenuity.force.com/ipa/articles/Feature_Description/Legend
(TIF)

Figure S2 **Adaptive immune response mega canonical pathway created using Ingenuity Pathway Analysis.** Please refer to Figure S5 for a legend explaining Pathway molecule symbols. For additional information, visit IPA legend help page at http://ingenuity.force.com/ipa/articles/Feature_Description/Legend
(TIF)

Figure S3 **Role of cytokines in mediating communication between immune cell canonical pathways from Ingenuity Pathway Analysis.** Please refer to Figure S5 for a legend explaining Pathway molecule symbols. For additional information, visit IPA legend help page at http://ingenuity.force.com/ipa/articles/Feature_Description/Legend
(TIF)

Figure S4 **Inhibition of matrix metalloproteases canonical pathway from Ingenuity Pathway Analysis.** MT-MMP complex includes *MMP15*, *MMP16*, and *MMP24*, among others, and these three were down-regulated in asymptomatic individuals. Extracellular MMP complex includes *MMP8*, *MMP10*, and *MMP12*, among others, and these three were also down-regulated. Please refer to Figure S5 for a legend explaining Pathway molecule symbols. For additional information, visit IPA legend help page at http://ingenuity.force.com/ipa/articles/Feature_Description/Legend
(TIF)

Figure S5 **Pathway molecule symbols legend from Ingenuity Pathway Analysis.** The color scheme used to indicate up- and down-regulation is different from that used by IPA and the intensity varies according to fold change. For additional information, visit IPA legend help page at http://ingenuity.force.com/ipa/articles/Feature_Description/Legend
(TIF)

Table S1 **53 of the selected canonical pathways studied, with genes up- and down-regulated in the subclinical/asymptomatic dengue are indicated.** Linear fold change (asymptomatic versus symptomatic) values are shown in bracket for those genes discussed in the manuscript. The pathways have been grouped according to the broad categories of reported dengue host defence mechanisms. The gene symbols are based on HUGO Gene Nomenclature Committee (HGNC) symbols.
(DOC)
