# Supplementary figures and images for: Lack of Clinical Manifestations in Asymptomatic Dengue Infection Is Attributed to Broad Down-Regulation and Selective Up-Regulation of Host Defence Response Genes
Source: PLoS One. 2014 Apr 11;9(4):e92240. doi: 10.1371/journal.pone.0092240 (PMC3984081; doi:10.1371/journal.pone.0092240)

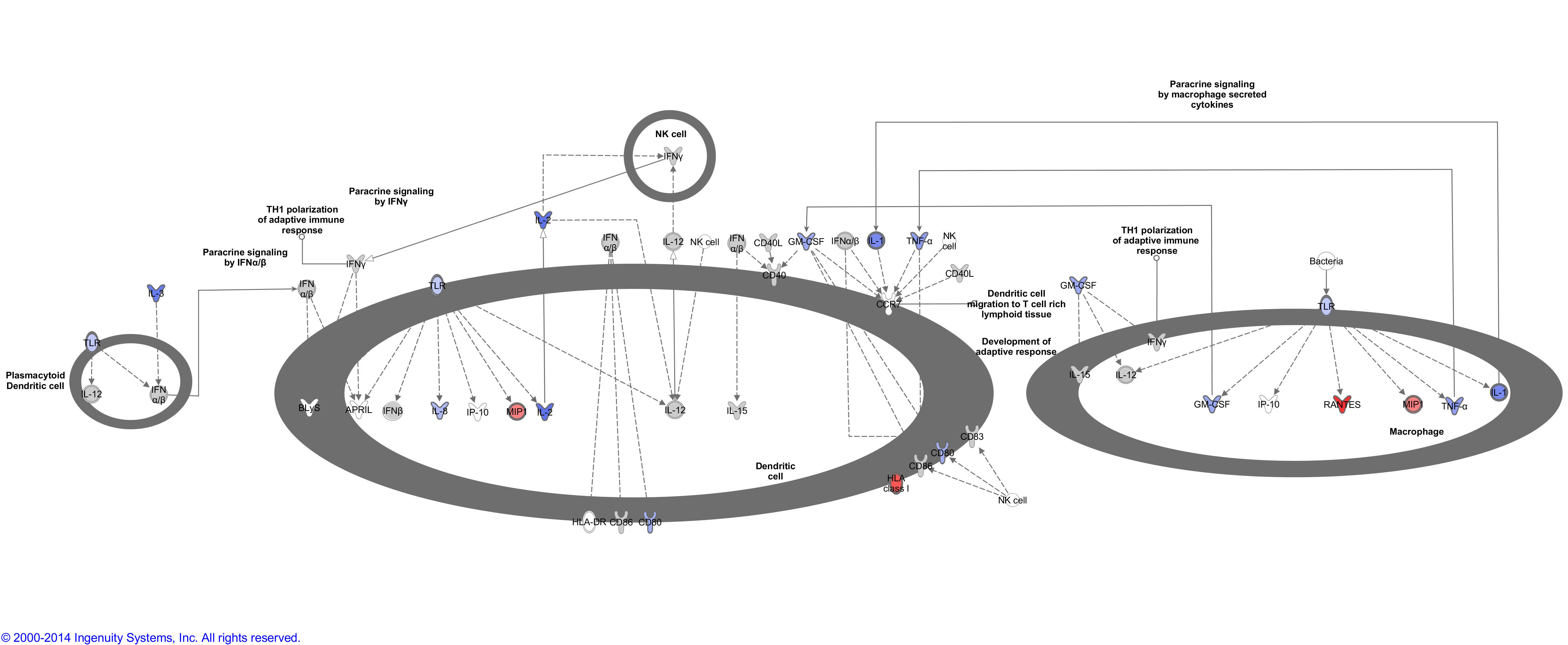

Supplement: Figure S1 — Innate immune response mega canonical pathway created using Ingenuity Pathway Analysis. TLR complex includes TLR6 (down-regulated in asymptomatic individuals), TLR3 (no change in expression between the two groups) and TLR7 (no change), among others. Please refer to Figure S5 for a legend explaining Pathway molecule symbols. For additional information, visit IPA legend help page at http://ingenuity.force.com/ipa/articles/Feature_Description/Legend (TIF) [file pone.0092240.s001.tif]

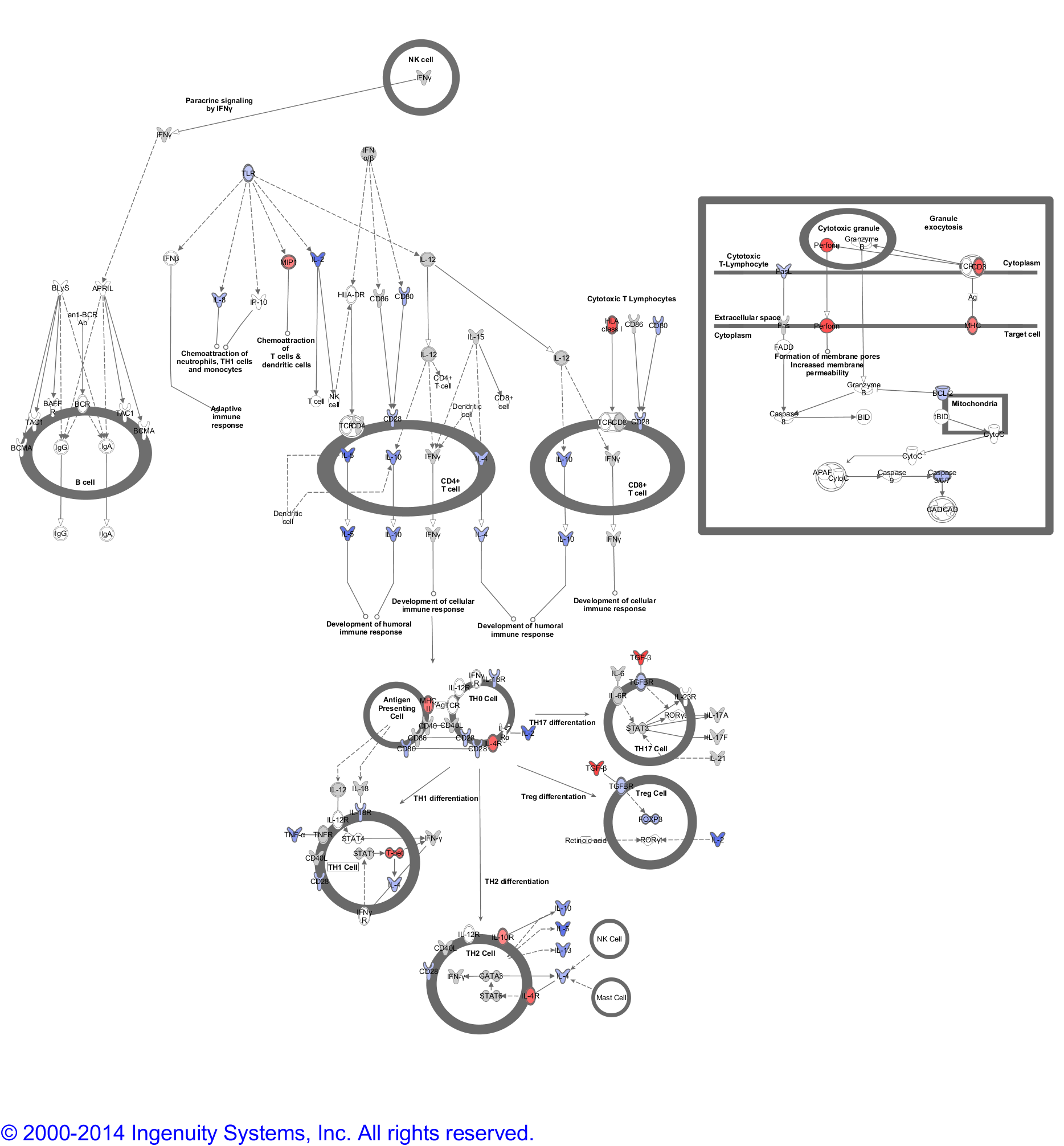

Supplement: Figure S2 — Adaptive immune response mega canonical pathway created using Ingenuity Pathway Analysis. Please refer to Figure S5 for a legend explaining Pathway molecule symbols. For additional information, visit IPA legend help page at http://ingenuity.force.com/ipa/articles/Feature_Description/Legend (TIF) [file pone.0092240.s002.tif]

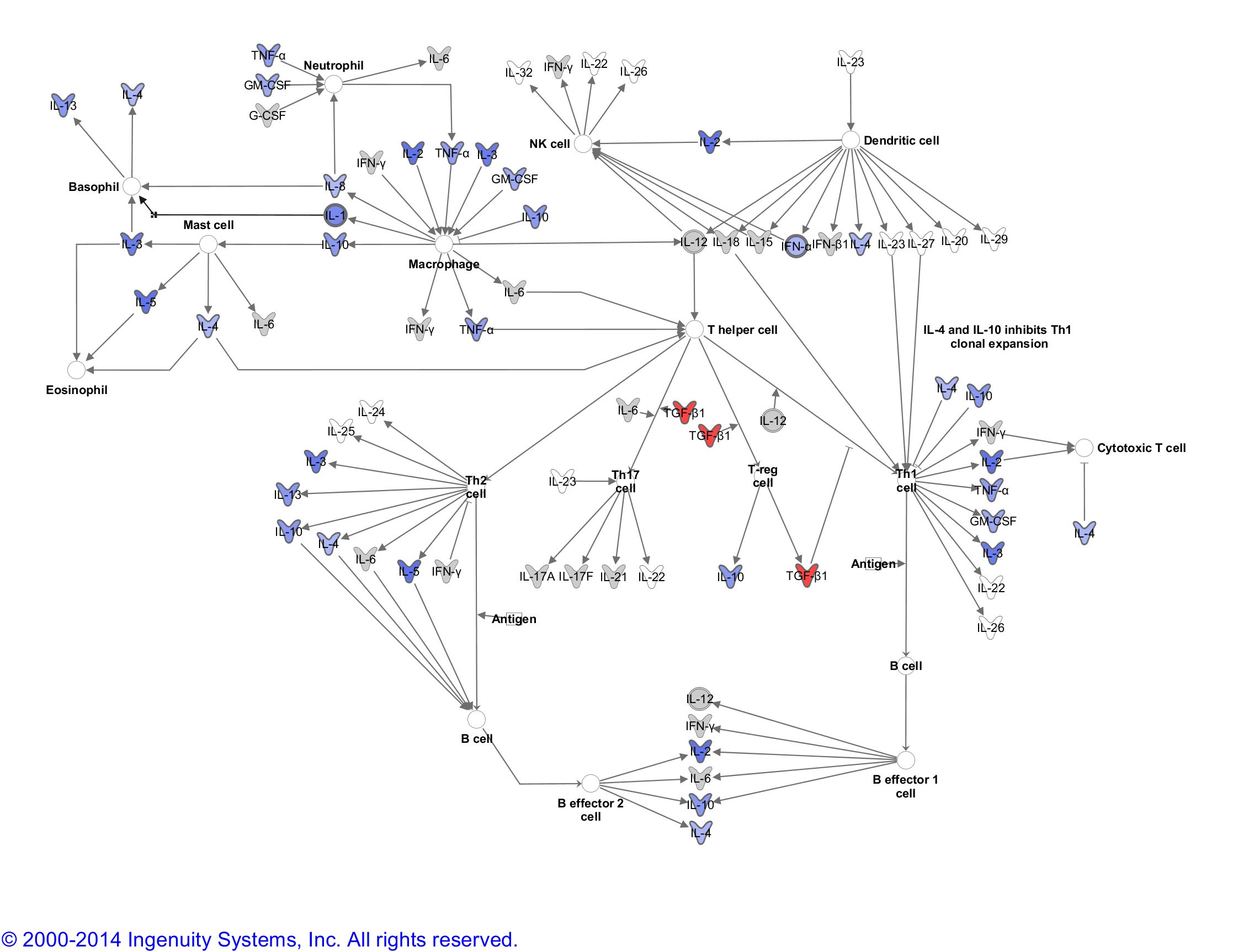

Supplement: Figure S3 — Role of cytokines in mediating communication between immune cell canonical pathways from Ingenuity Pathway Analysis. Please refer to Figure S5 for a legend explaining Pathway molecule symbols. For additional information, visit IPA legend help page at http://ingenuity.force.com/ipa/articles/Feature_Description/Legend (TIF) [file pone.0092240.s003.tif]

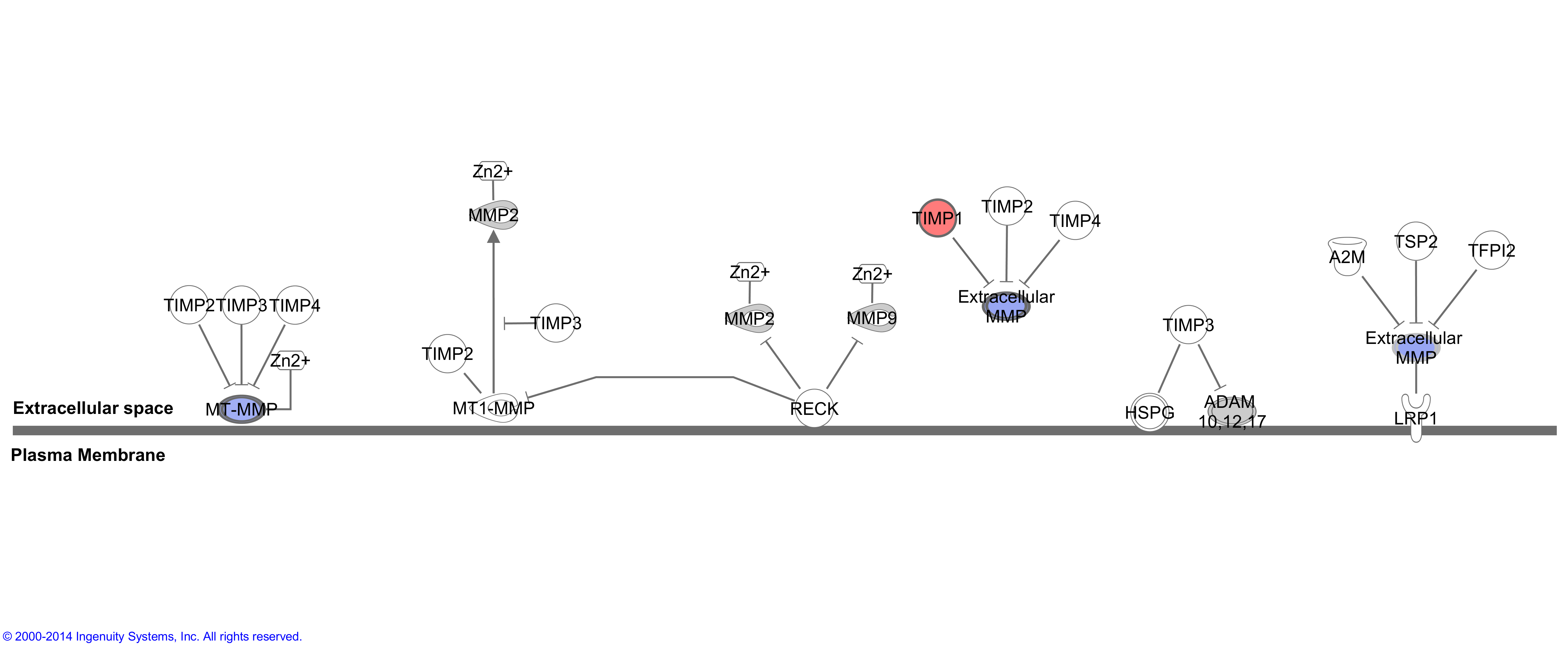

Supplement: Figure S4 — Inhibition of matrix metalloproteases canonical pathway from Ingenuity Pathway Analysis. MT-MMP complex includes MMP15, MMP16, and MMP24, among others, and these three were down-regulated in asymptomatic individuals. Extracellular MMP complex includes MMP8, MMP10, and MMP12, among others, and these three were also down-regulated. Please refer to Figure S5 for a legend explaining Pathway molecule symbols. For additional information, visit IPA legend help page at http://ingenuity.force.com/ipa/articles/Feature_Description/Legend (TIF) [file pone.0092240.s004.tif]

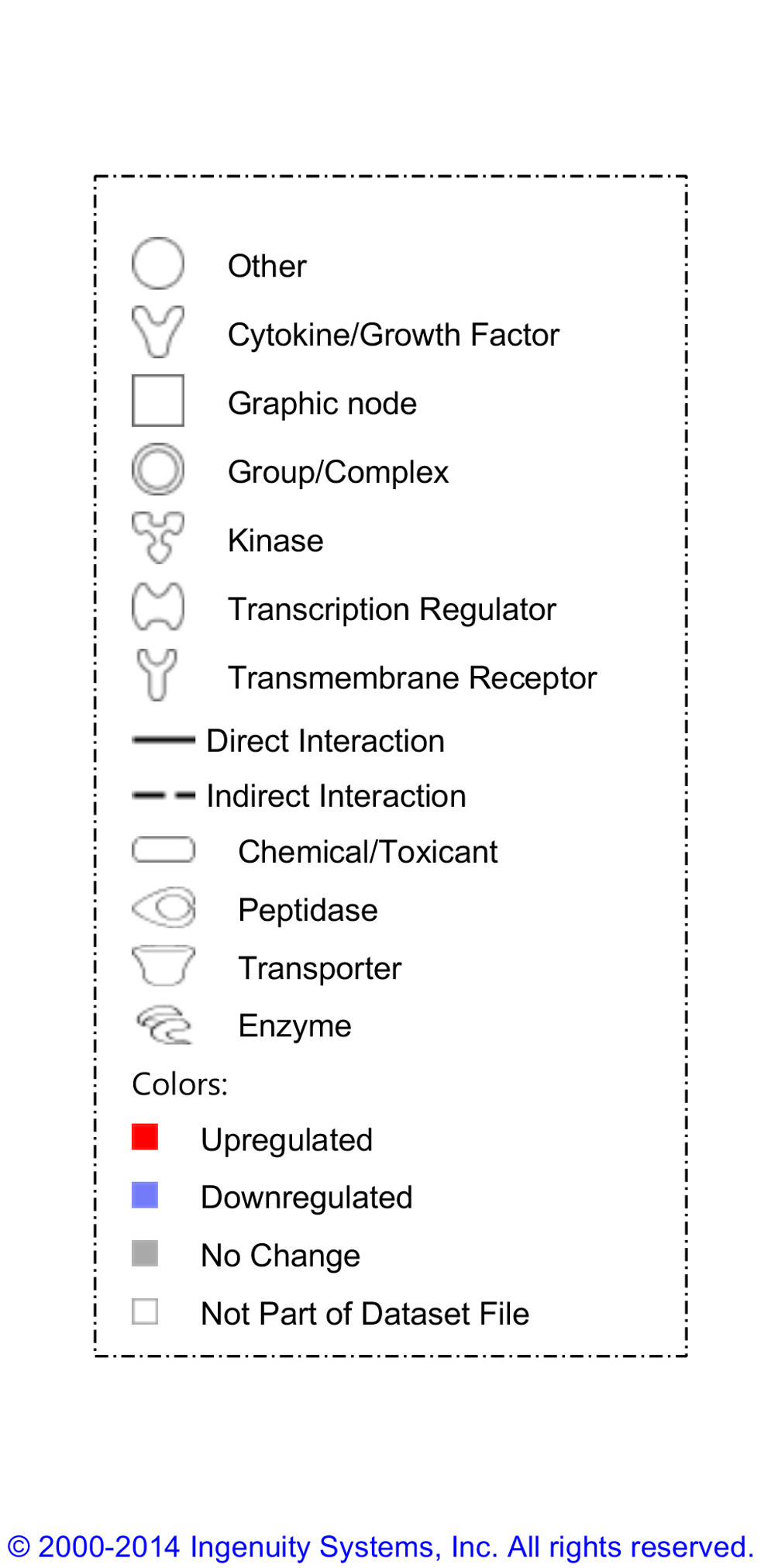

Supplement: Figure S5 — Pathway molecule symbols legend from Ingenuity Pathway Analysis. The color scheme used to indicate up- and down-regulation is different from that used by IPA and the intensity varies according to fold change. For additional information, visit IPA legend help page at http://ingenuity.force.com/ipa/articles/Feature_Description/Legend (TIF) [file pone.0092240.s005.tif]
