# Supplementary material for: Lack of Clinical Manifestations in Asymptomatic Dengue Infection Is Attributed to Broad Down-Regulation and Selective Up-Regulation of Host Defence Response Genes
Source: PLoS One. 2014 Apr 11;9(4):e92240. doi: 10.1371/journal.pone.0092240 (PMC3984081; doi:10.1371/journal.pone.0092240)
Supplement: Table S1 — 53 of the selected canonical pathways studied, with genes up- and down-regulated in the subclinical/asymptomatic dengue are indicated. Linear fold change (asymptomatic versus symptomatic) values are shown in bracket for those genes discussed in the manuscript. The pathways have been grouped according to the broad categories of reported dengue host defence mechanisms. The gene symbols are based on HUGO Gene Nomenclature Committee (HGNC) symbols. (DOC) [file pone.0092240.s006.doc]

Table S1

| **Group** | **Canonical Pathway** | | **Differential Expression** | | |
| --- | --- | --- | --- | --- | --- |
|  | **No.** | **Name** | **Up-regulation** | **Down-regulation** | **No change** |
| Innate | 1 | Acute phase response signaling | AKT1, FOS, IKBKG, NFKB1, NFKBIA, TRADD | C1S (-2.611), CFB (-2.009), CRP, IL1A (-4.629), IL1RAP, MAP2K7, SOCS3, TNF (-3.767) | AMBP, CEBPB, CHUK, FN1, GRB2, HMOX1, HRAS, IKBKB, IL18 (-1.602), IL1B (-1.415), IL1R1, IL1RN, IL6 (-1.367), IL6R, JAK2, JUN, MAP2K1, MAP3K7, MAPK1, MAPK14, MTOR, MYD88, NFKB2, NFKBIB, NR3C1, PIK3R1, RELA, SERPINA1, SOCS1, SOD2, STAT3, TCF3, TCF4, TNFRSF1A, TRAF2, TRAF6 |
|  | 2 | CCR5 signaling in macrophages | CALM1, CCL5 (3.393), CD247, CD3D, CD3E, FOS, PRKCB | FASLG, PLCG1, PRKCE | CCR5 (1.983), CD4 (1.038), FAS, JUN, MAPK1, MAPK14, PRKCA, PRKCD, PRKCH |
|  | 3 | CD27 signaling in lymphocytes | FOS, IKBKG, NFKB1, NFKBIA | MAP2K7, MAP3K2, MAP3K4 | CHUK, IKBKB, JUN, MAP2K1, MAP3K7, MAP3K8, NFKB2, NFKBIB, RELA, TRAF2 |
|  | 4 | CD40 signaling | FOS, IKBKG, NFKB1, NFKBIA | ICAM1, LTA, MAP2K7, PIK3C2A, PIK3C2G | ATF1, CD40 (1.408), CD40LG, CHUK, FCER2, IKBKB, JAK3, JUN, MAP2K1, MAP3K7, MAPK1, MAPK14, MAPKAPK2, NFKB2, NFKBIB, PIK3R1, RELA, STAT3, TANK, TRAF2, TRAF6 |
|  | 5 | Complement system |  | C1S (-2.611), CFB (-2.009) | C5AR1, CD46, CFH |
|  | 6 | Crosstalk between dendritic cells and natural killer cells | ACTB, ICAM3, IL2RG, KLRC4-KLRK1/KLRK1, KLRD1, LTB, NFKB1, PRF1 | CD28 (-2.437), CD80 (-3.116), CSF2 (-3.353), CSF2RB, FASLG, IL2 (-6.664), IL2RB, IL3 (-5.124), IL4 (-2.842), LTA, LTBR, TNF (-3.767) | CD40 (1.408), CD40LG, CD69, CD83 (-1.724), CD86 (-1.13), FAS, IFNB1 (-1.111), IFNG (-1.747), IL12B (-1.264), IL15 (1.471), IL18 (-1.602), IL3RA, IL6 (-1.367), ITGAL, NFKB2, RELA, TLR3 (-1.546), TLR4 (1.392), TLR7 (1.465), TLR9 (1.094), TNFSF10 |
|  | 7 | Dendritic cell maturation | AKT1, B2M (2.807), CD1D, FCGR3A (2.126), HLA-D-QA1 (2.213), IKBKG, LTB, NFKB1, NFKBIA | CD1A, CD1B, CD80 (-3.116), COL11A2, COL1A1, COL1A2, COL2A1, COL3A1, CSF2 (-3.353), ICAM1, IL10 (-4.005), IL1A (-4.629), LTA, LTBR, LY75, PIK3C2A, PIK3C2G, PLCB1, PLCB4, PLCD1, PLCD3, PLCE1, PLCG1, TNF (-3.767) | ATF2, CD40 (1.408), CD40LG, CD83 (-1.724), CD86 (-1.13), CHUK, COL18A1, CREB1, DDR1, FCGR1A (1.023), FCGR2B (1.295), IFNAR1, IFNB1 (-1.111), IKBKB, IL12B (-1.264), IL15 (1.471), IL18 (-1.602), IL1B (-1.415), IL1RN, IL6 (-1.367), JAK2, MAPK1, MAPK14, MYD88, NFKB2, NFKBIB, PIK3R1, PLCL1, RELA, RELB, STAT1, STAT2, TLR2 (1.343), TLR3 (-1.546), TLR4 (1.392), TLR9 (1.094), TNFRSF1A, TRAF6 |
|  | 8 | iNOS signaling | CALM1, FOS, IKBKG, IRF1, NFKB1, NFKBIA | - | CD14, CHUK, IFNG (-1.747), IKBKB, IRAK2, IRAK4, JAK1, JAK2, JAK3, JUN, MAPK1, MAPK14, MYD88, NFKB2, NFKBIB, RELA, STAT1, TLR4 (1.392), TRAF6 |
|  | 9 | Mechanisms of viral exit from host cells | ACTB, PRKCB | NEDD4, PRKCE | PRKCA, PRKCD, PRKCH |
|  | 10 | MIF regulation of innate immunity | CD74, FOS, NFKB1, NFKBIA | PLA2G12B, PLA2G3, PLA2G4A | CD14, JUN, MAPK1, NFKB2, NFKBIB, PTGS2, RELA, TLR4 (1.392), TP53 |
|  | 11 | Natural killer cell signaling | AKT1, CD247, FCGR3A (2.126), FYN, KLRC4-KLRK1/KLRK1, KLRD1, LCK, PRKCB | PIK3C2A, PIK3C2G, PLCG1, PRKCE, VAV3 | GRB2, HRAS, MAP2K1, MAPK1, PIK3R1, PRKCA, PRKCD, PRKCH, SYK, VAV1, ZAP70 |
|  | 12 | Production of Nitric Oxide and reactive Oxygen species in macrophages | AKT1, CLU, FOS, IKBKG, IRF1, NFKB1, NFKBIA, PRKCB, RHOA | IL4 (-2.842), MAP2K7, MAP3K2, MAP3K4, PIK3C2A, PIK3C2G, PLCG1, PPARA, PRKCE, TNF (-3.767) | CHUK, IFNG (-1.747), IKBKB, JAK1, JAK2, JAK3, JUN, MAP2K1, MAP3K7, MAP3K8, MAPK1, MAPK14, NFKB2, NFKBIB, PIK3R1, PRKCA, PRKCD, PRKCH, RELA, RHOB, SERPINA1, STAT1, TLR2 (1.343), TLR4 (1.392), TNFRSF1A |
|  | 13 | Role of pattern recognition receptors in recognition of bacteria and viruses | CASP1, CCL5 (3.393), NFKB1, PRKCB | IL10 (-4.005), IL2 (-6.664), PIK3C2A, PIK3C2G, PRKCE, TLR6 (-2.113), TNF (-3.767) | C5AR1, CREB1, IFNB1 (-1.111), IL12B (-1.264), IL1B (-1.415), IL6 (-1.367), IRF3, IRF7, MAPK1, MYD88, NFKB2, NOD2, PIK3R1, PRKCA, PRKCD, PRKCH, RELA, SYK, TLR1, TLR2 (1.343), TLR3 (-1.546), TLR4 (1.392), TLR5, TLR7 (1.465), TLR9 (1.094), TRAF6 |
|  | 14 | Role of PKR in interferon induction and antiviral response | AKT1, IKBKG, IRF1, NFKB1, NFKBIA | TNF (-3.767) | ATF2, CHUK, FCGR1A (1.023), IFNB1 (-1.111), IFNG (-1.747), IKBKB, MAP3K7, MAPK14, NFKB2, NFKBIB, RELA, STAT1, TLR3 (-1.546), TNFRSF1A, TP53, TRAF2, TRAF6 |
|  | 15 | Role of RIG1-like receptors in antiviral innate immunity | IKBKG, NFKB1, NFKBIA | CASP10, IFNA2 (-2.858) | CHUK, IFNB1 (-1.111), IKBKB, IRF3, IRF7, NFKB2, NFKBIB, RELA, TANK, TRAF2, TRAF6 |
|  | 16 | Toll-like receptor signaling | FOS, IKBKG, NFKB1, NFKBIA | PPARA, TLR6 (-2.113) | CD14, CHUK, IKBKB, IRAK2, IRAK4, JUN, MAP3K7, MAPK1, MAPK14, MYD88, NFKB2, RELA, TIRAP, TLR1, TLR2 (1.343), TLR3 (-1.546), TLR4 (1.392), TLR5, TLR7 (1.465), TLR9 (1.094), TRAF6 |
|  | 17 | TWEAK signaling | IKBKG, NFKB1, NFKBIA, TNFRSF25, TRADD | CASP7 | BIRC2, BIRC3, CHUK, IKBKB, NFKB2, NFKBIB, RELA, TNFSF12, TRAF2 |
| Adaptive | 18 | B cell development | HLA-D-QA1 (2.213), IL7R (2.73), PTPRC | CD80 (-3.116), RAG1 | CD40 (1.408), CD86 (-1.13), IL7 (-1.663) |
|  | 19 | B cell receptor signaling | AKT1, CALM1, CSK, IKBKG, NFATC3, NFKB1, NFKBIA, PRKCB, PTEN, PTPRC | EGR1, MAP2K7, MAP3K2, MAP3K4, PIK3C2A, PIK3C2G, VAV3 | ATF2, BAD, BCL10, BCL6, BTK, CHUK, CREB1, ETS1, FCGR2B (1.295), FOXO1, GRB2, HRAS, IKBKB, JUN, MAP2K1, MAP3K7, MAP3K8, MAPK1, MAPK14, MTOR, NFATC2, NFKB2, NFKBIB, PIK3R1, RELA, SYK, TCF3, VAV1 |
|  | 20 | B cell activating factor signaling | FOS, IKBKG, NFATC3, NFKB1, NFKBIA | MAP2K7 | CHUK, IKBKB, JUN, MAPK1, MAPK14, NFATC2, NFKB2, NFKBIB, RELA, TRAF2, TRAF6 |
|  | 21 | CD28 signaling in T helper cells | AKT1, CALM1, CD247, CD3D, CD3E, CSK, FOS, FYN, HLA-D-QA1 (2.213), IKBKG, ITK, LCK, NFATC3, NFKB1, NFKBIA, PTPRC | CD28 (-2.437), CD80 (-3.116), IL2 (-6.664), PIK3C2A, PIK3C2G, PLCG1 | BCL10, CD4 (1.038), CD86 (-1.13), CHUK, GRB2, IKBKB, JUN, MAP2K1, NFATC2, NFKB2, NFKBIB, PIK3R1, RELA, SYK, VAV1, ZAP70 |
|  | 22 | CTLA4 signaling in cytotoxic T lymphocytes | AKT1, B2M (2.807), CD247, CD3D, CD3E, FYN, LCK | CD28 (-2.437), CD80 (-3.116), PIK3C2A, PIK3C2G, PLCG1 | CD86 (-1.13), GRB2, JAK2, PIK3R1, SYK, ZAP70 |
|  | 23 | Cytotoxic T lymphocytes-mediated apoptosis of target cells | B2M (2.807), CD247, CD3D, CD3E, HLA-D-QA1 (2.213), PRF1 | BCL2, CASP7, FASLG | FAS |
|  | 24 | Differential regulation of cytokine production in intestinal epithelial cells by IL-17A and IL-18 | CCL5 (3.393) | CSF2 (-3.353), CXCL1, IL3 (-5.124), IL9 (-4.505), IL10 (-4.005), IL13 (-4.059), IL1A (-4.629), TNF (-3.767) | CCL2 (1.284), CSF3, IFNG (-1.747), IL12B (-1.264), IL17A (-1.158), IL17F (-1.958), IL1B (-1.415), LCN2, CCL2 (1.284), CSF3, IL6 (-1.367), IL12B (-1.264), IL17A (-1.158), IL17F (-1.958), IL1B (-1.415) |
|  | 25 | Differential regulation of cytokine production in macrophages and T helper cells by IL-17A and IL-18 | CCL5 (3.393) | CSF2 (-3.353), CXCL1, IL3 (-5.124), IL9 (-4.505), IL10 (-4.005), IL13 (-4.059), TNF (-3.767) | - |
|  | 26 | Fcγ Receptor-mediated Phagocytosis in Macrophages and Monocytes | ACTB, AKT1, FCGR3A (2.126), FGR, FYN, PRKCB, PTEN | CBL, CSF2 (-3.353), PIK3C2G, PLCG1, PRKCE, VAV3, YES1 | FCGR1A (1.023), HMOX1, MAPK1, PIK3R1, PRKCA, PRKCD, PRKCH, SRC, SYK, VAV1 |
|  | 27 | FcγRIIB Signaling in B Lymphocytes | AKT1 | PIK3C2A, PIK3C2G | BTK, FCGR2B (1.295), GRB2, HRAS, PIK3R1, SYK |
|  | 28 | NF-kB activation by viruses | AKT1, IKBKG, ITGB1, ITGB2, LCK, NFKB1, NFKBIA, PRKCB | ITGA2, ITGAV, PIK3C2A, PIK3C2G, PRKCE | CCR5 (1.983), CD4 (1.038), CHUK, HRAS, IKBKB, ITGA1, ITGA3, ITGA4, ITGA5, ITGAL, ITGB3, MAPK1, NFKB2, NFKBIB, PIK3R1, PRKCA, PRKCD, PRKCH, RELA, TRAF2 |
|  | 29 | T cell receptor signaling | CALM1, CD247, CD3D, CD3E, CSK, FOS, FYN, IKBKG, ITK, LCK, NFATC3, NFKB1, NFKBIA, PTPRC | CBL, CD28 (-2.437), PIK3C2A, PIK3C2G, PLCG1, VAV3 | BCL10, BTK, CD4 (1.038), CHUK, GRB2, HRAS, IKBKB, JUN, MAP2K1, MAPK1, NFATC2, NFKB2, PIK3R1, RELA, VAV1, ZAP70 |
|  | 30 | T helper cell differentiation | HLA-D-QA1 (2.213), IL10RA (2.001), IL2RG, TBX21 (2.622), TGFB1 (3.108) | CD28 (-2.437), CD80 (-3.116), FOXP3 (-2.877), IL10 (-4.005), IL13 (-4.059), IL18R1 (-2.646), IL2 (-6.664), IL4 (-2.842), IL5 (-7.894), TGFBR1 (-2.257), TNF (-3.767) | BCL6, CD40 (1.408), CD40LG, CD86 (-1.13), GATA3, ICOS, ICOSLG, IFNG (-1.747), IL12B (-1.264), IL17A (-1.158), IL17F (-1.958), IL18 (-1.602), IL21 (-1.398), IL6 (-1.367), IL6 (-1.367)R, STAT1, STAT3, STAT6, TNFRSF1A |
| Cytokine | 31 | Chemokine signaling | CALM1, CCL5 (3.393), CXCR4, FOS, MPRIP, PRKCB, RHOA | PIK3C2G, PLCB1, PLCB4, PLCG1 | CCL2 (1.284), CCR5 (1.983), CXCL12, HRAS, JUN, MAP2K1, MAPK1, MAPK14, PRKCA, SRC |
|  | 32 | GM-CSF signaling | AKT1, PRKCB | CCND1, CSF2 (-3.353), CSF2RA, CSF2RB, PIK3C2A, PIK3C2G | ETS1, GRB2, HRAS, JAK2, MAP2K1, MAPK1, PIK3R1, STAT1, STAT3, STAT5B |
|  | 33 | IL-1 signaling | FOS, IKBKG, NFKB1, NFKBIA | ADCY8, IL1A (-4.629), IL1RAP, MAP2K7 | CHUK, IKBKB, IL1R1, IRAK2, IRAK4, JUN, MAP3K7, MAPK1, MAPK14, MYD88, NFKB2, NFKBIB, RELA, TRAF6 |
|  | 34 | IL-10 signaling | FOS, IKBKG, IL10RA (2.001), NFKB1, NFKBIA | IL10 (-4.005), IL1A (-4.629), IL1RAP, IL1RAPL1, IL1RAPL2, SOCS3, TNF (-3.767) | CCR5 (1.983), CD14, CHUK, FCGR2B (1.295), HMOX1, IKBKB, IL18 (-1.602), IL1B (-1.415), IL1R1, IL1RL1, IL1RN, IL6 (-1.367), JAK1, JUN, MAP3K7, MAPK1, MAPK14, NFKB2, NFKBIB, RELA, SP1, STAT3, TRAF6 |
|  | 35 | IL-15 production | IRF1, NFKB1 | MST1R | IFNB1 (-1.111), IL15 (1.471), IL6 (-1.367), IRF3, JAK1, JAK2, JAK3, NFKB2, RELA, STAT1 |
|  | 36 | IL-15 signaling | AKT1, IL2RG, LCK, NFKB1, STAT5A | BCL2, CSF2 (-3.353), IL2RB, IL4 (-2.842), PIK3C2A, PIK3C2G, PLCG1 | HRAS, IL15 (1.471), IL17A (-1.158), JAK1, JAK2, JAK3, MAP2K1, MAPK1, MAPK14, NFKB2, PIK3R1, RELA, STAT3, STAT5B, STAT6, SYK, TRAF2 |
|  | 37 | IL-2 signaling | AKT1, FOS, IL2RG, LCK, STAT5A | IL2 (-6.664), IL2RB, PIK3C2A, PIK3C2G | GRB2, HRAS, JAK1, JAK3, JUN, MAP2K1, MAPK1, PIK3R1, SOCS1, STAT5B, SYK |
|  | 38 | IL-22 signaling | AKT1, STAT5A | IL22RA1 (-2.93), SOCS3 | JAK1, MAPK1, MAPK14, STAT1, STAT3, STAT5B |
|  | 39 | IL-3 signaling | AKT1, FOS, PRKCB, STAT5A | CRKL, CSF2RB, IL3 (-5.124), PIK3C2A, PIK3C2G, PRKCE | BAD, FOXO1, GRB2, HRAS, IL3RA, JAK1, JAK2, JUN, MAP2K1, MAPK1, PIK3R1, PRKCA, PRKCD, PRKCH, STAT1, STAT3, STAT5B, STAT6 |
|  | 40 | IL-4 signaling | AKT1, HLA-D-QA1 (2.213), IL2RG, NFATC3 | IL4 (-2.842), PIK3C2A, PIK3C2G | FCER2, GRB2, HRAS, JAK1, JAK2, JAK3, MTOR, NFATC2, NR3C1, PIK3R1, SOCS1, STAT6 |
|  | 41 | IL-6 signaling | AKT1, FOS, IKBKG, NFKB1, NFKBIA | COL1A1, CRP, IL1A (-4.629), IL1RAP, IL1RAPL1, IL1RAPL2, IL8 (-2.72), MAP2K7, PIK3C2A, PIK3C2G, SOCS3, SRF, TNF (-3.767) | CD14, CEBPB, CHUK, GRB2, HRAS, IKBKB, IL18 (-1.602), IL1B (-1.415), IL1R1, IL1RL1, IL1RN, IL6 (-1.367), IL6R, JAK2, JUN, MAP2K1, MAP3K7, MAPK1, MAPK14, MAPKAPK2, NFKB2, NFKBIB, PIK3R1, RELA, SOCS1, STAT3, TNFRSF1A, TRAF2, TRAF6, VEGFA (1.39) |
|  | 42 | IL-8 signaling | AKT1, FOS, IKBKG, ITGAX, ITGB2, NFKB1, PRKCB, RHOA | BCL2, CCND1, CXCL1, FIGF, ICAM1, IL8 (-2.72), IL9 (-4.505), ITGAV, KDR (-2.002), PGF, PIK3C2A, PIK3C2G, PRKCE, VEGFC (-2.118) | CHUK, CXCR1, HMOX1, HRAS, IKBKB, IQGAP1, IRAK2, IRAK4, ITGAM, ITGB3, JUN, MAP2K1, MAPK1, MMP2 (-1.858), MMP9 (-1.328), MTOR, NFKBIB, PIK3R1, PRKCA, PRKCD, PRKCH, PTGS2, RELA, RHOB, SRC, TRAF6, VCAM1, VEGFA (1.39) |
|  | 43 | IL-9 signaling | IL2RG, NFKB1, STAT5A | IL9 (-4.505), PIK3C2A, PIK3C2G, SOCS3, TNF (-3.767) | BCL3, JAK1, JAK3, NFKB2, PIK3R1, RELA, STAT1, STAT3, STAT5B |
| MMP | 44 | Inhibition of matrix metalloproteases | TIMP1 (2.193) | MMP10 (-3.525), MMP12 (-3.514), MMP15 (-3.204), MMP16 (-2.871), MMP24 (-2.525), MMP8 (-2.879) | ADAM17, MMP2 (-1.858), MMP9 (-1.328) |
| Interaction between innate, cytokines and adaptive | 45 | TREM1 signaling | AKT1, CASP1, ITGAX, ITGB1, NFKB1, STAT5A | CSF2 (-3.353), CXCL3 (-2.364), ICAM1, IL10 (-4.005), IL8 (-2.72), PLCG1, TLR6 (-2.113), TNF (-3.767) | CCL2 (1.284), CD40 (1.408), CD83 (-1.724), CD86 (-1.13), FCGR2B (1.295), GRB2, IL18 (-1.602), IL1B (-1.415), IL6 (-1.367), ITGA5, JAK2, MAPK1, MYD88, NFKB2, NOD2, RELA, STAT3, STAT5B, TLR1, TLR2 (1.343), TLR3 (-1.546), TLR4 (1.392), TLR5, TLR7 (1.465), TLR9 (1.094) |
|  | 46 | Interferon signaling | IRF1, PTPN2, TAP1 | BCL2, IFNAR2 | IFNAR1, IFNB1 (-1.111), IFNG (-1.747), JAK1, JAK2, RELA, SOCS1, STAT1, STAT2 |
|  | 47 | IL-17 signaling | AKT1, IL17RA, NFKB1, TIMP1 (2.193) | CRP, CXCL1, IL19, IL8 (-2.72), PIK3C2A, PIK3C2G | ATF2, CCL2 (1.284), CEBPB, HRAS, IL17A (-1.158), IL17F (-1.958), IL6 (-1.367), JAK1, JAK2, JUN, MAP2K1, MAP3K7, MAPK1, MAPK14, MAPKAPK2, PIK3R1, PTGS2, RELA, TRAF6 |
|  | 48 | IL-17A signaling in fibroblasts | FOS, IKBKG, IL17RA, NFKB1, NFKBIA |  | CCL2 (1.284), CEBPB, CHUK, IKBKB, IL17A (-1.158), IL6 (-1.367), JUN, LCN2, MAP3K7, MAPK1, MAPK14, NFKB2, NFKBIB, RELA, TRAF6 |
|  | 49 | Regulation of IL-2 expression in activated and anergic T lymphocytes | CALM1, CD247, CD3D, CD3E, FOS, FYN, IKBKG, NFATC3, NFKB1, NFKBIA, TGFB1 (3.108) | CD28 (-2.437), CD80 (-3.116), IL2 (-6.664), MAP2K7, PLCG1, TGFB2 (-2.237), TGFBR1 (-2.257), VAV3 | BCL10, CHUK, GRB2, HRAS, IKBKB, JUN, MAP2K1, MAPK1, NFATC2, NFKB2, NFKBIB, RELA, VAV1, ZAP70 |
|  | 50 | TGF-β Signaling | BMP4, FOS, TGFB1 (3.108) | BCL2, BMP2, BMP7, FOXH1, PIAS4, TGFB2 (-2.237), TGFBR1 (-2.257) | GRB2, HRAS, INHBA, IRF7, JUN, MAP2K1, MAP3K7, MAPK1, MAPK14, SMAD1, TRAF6 |
|  | 51 | Communication between innate and adaptive immune cells | B2M (2.807), CCL3L1/CCL3L3 (2.097), CCL5 (3.393) | CD28 (-2.437), CD80 (-3.116), CSF2 (-3.353), IL10 (-4.005), IL1A (-4.629), IL2 (-6.664), IL3 (-5.124), IL4 (-2.842), IL5 (-7.894), IL8 (-2.72), TLR6 (-2.113), TNF (-3.767) | CD4 (1.038), CD8A (-1.259), CD8B (-1.862), CD40 (1.408), CD40LG, CD83 (-1.724), CD86 (-1.13), IFNB1 (-1.111), IFNG (-1.747), IL12B (-1.264), IL15 (1.471), IL18 (-1.602), IL1B (-1.415), IL1RN, IL6 (-1.367), TLR1, TLR2 (1.343), TLR3 (-1.546), TLR4 (1.392), TLR5, TLR7 (1.465), TLR9 (1.094) |
|  | 52 | IL-12 signaling and production in macrophages | AKT1, CLU, FOS, IKBKG, IRF1, NFKB1, PRKCB, TGFB1 (3.108) | IFNA2 (-2.858), IL10 (-4.005), IL4 (-2.842), MST1R, PIK3C2A, PIK3C2G, PRKCE, TGFB2 (-2.237), TNF (-3.767) | CD40 (1.408), CD40LG, CEBPB, CHUK, IFNG (-1.747), IKBKB, IL12B (-1.264), IL18 (-1.602), JUN, MAF, MAP2K1, MAP3K8, MAPK1, MAPK14, MYD88, NFKB2, NFKBIB, PIK3R1, PRKCA, PRKCD, PRKCH, REL, RELA, SERPINA1, STAT1, STAT6, TLR2 (1.343), TLR4 (1.392), TRAF6 |
|  | 53 | Role of cytokines in mediating communication between immune cells | TGFB1 (3.108) | CSF2 (-3.353), IFNA2 (-2.858), IL10 (-4.005), IL13 (-4.059), IL1A (-4.629), IL2 (-6.664), IL3 (-5.124), IL4 (-2.842), IL5 (-7.894), IL8 (-2.72), TNF (-3.767) | CSF3, IFNB1 (-1.111), IFNG (-1.747), IL12B (-1.264), IL15 (1.471), IL17A (-1.158), IL17F (-1.958), IL18 (-1.602), IL1B (-1.415), IL1RN, IL21 (-1.398), IL6 (-1.367) |
